# A KDPG sensor RccR governs *Pseudomonas aeruginosa* carbon metabolism and aminoglycoside antibiotic tolerance

**DOI:** 10.1093/nar/gkad1201

**Published:** 2023-12-14

**Authors:** Yujue Wang, Zhipeng Wang, Weizhong Chen, Ze-Hui Ren, Hui Gao, Jiani Dai, Guan-Zheng Luo, Zhaowei Wu, Quanjiang Ji

**Affiliations:** School of Physical Science and Technology, ShanghaiTech University, Shanghai 201210, China; School of Physical Science and Technology, ShanghaiTech University, Shanghai 201210, China; School of Physical Science and Technology, ShanghaiTech University, Shanghai 201210, China; MOE Key Laboratory of Gene Function and Regulation, Guangdong Province Key Laboratory of Pharmaceutical Functional Genes, State Key Laboratory of Biocontrol, School of Life Sciences, Sun Yat-sen University, Guangzhou 510275, Guangdong, China; School of Physical Science and Technology, ShanghaiTech University, Shanghai 201210, China; School of Physical Science and Technology, ShanghaiTech University, Shanghai 201210, China; MOE Key Laboratory of Gene Function and Regulation, Guangdong Province Key Laboratory of Pharmaceutical Functional Genes, State Key Laboratory of Biocontrol, School of Life Sciences, Sun Yat-sen University, Guangzhou 510275, Guangdong, China; School of Physical Science and Technology, ShanghaiTech University, Shanghai 201210, China; School of Physical Science and Technology, ShanghaiTech University, Shanghai 201210, China; Gene Editing Center, School of Life Science and Technology, ShanghaiTech University, Shanghai 201210, China; Shanghai Clinical Research and Trial Center, Shanghai 201210, China

## Abstract

*Pseudomonas aeruginosa* harbors sophisticated transcription factor (TF) networks to coordinately regulate cellular metabolic states for rapidly adapting to changing environments. The extraordinary capacity in fine-tuning the metabolic states enables its success in tolerance to antibiotics and evading host immune defenses. However, the linkage among transcriptional regulation, metabolic states and antibiotic tolerance in *P. aeruginosa* remains largely unclear. By screening the *P. aeruginosa* TF mutant library constructed by CRISPR/Cas12k-guided transposase, we identify that *rccR* (PA5438) is a major genetic determinant in aminoglycoside antibiotic tolerance, the deletion of which substantially enhances bacterial tolerance. We further reveal the inhibitory roles of RccR in pyruvate metabolism (*aceE*/*F*) and glyoxylate shunt pathway (*aceA* and *glcB*), and overexpression of *aceA* or *glcB* enhances bacterial tolerance. Moreover, we identify that 2-keto-3-deoxy-6-phosphogluconate (KDPG) is a signal molecule that directly binds to RccR. Structural analysis of the RccR/KDPG complex reveals the detailed interactions. Substitution of the key residue R152, K270 or R277 with alanine abolishes KDPG sensing by RccR and impairs bacterial growth with glycerol or glucose as the sole carbon source. Collectively, our study unveils the connection between aminoglycoside antibiotic tolerance and RccR-mediated central carbon metabolism regulation in *P*. *aeruginosa*, and elucidates the KDPG-sensing mechanism by RccR.

## Graphical Abstract

**Graphical Abstract:**
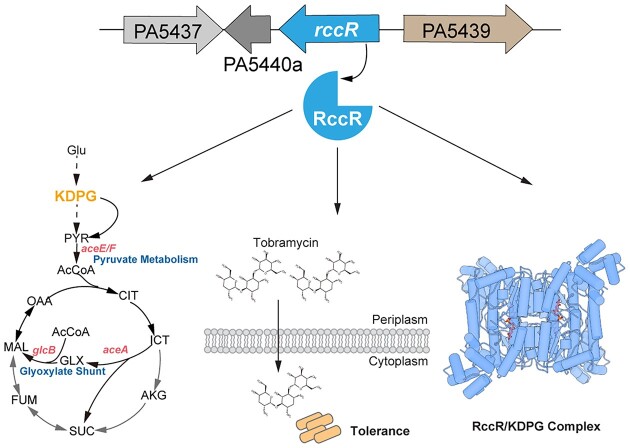

## Introduction

*Pseudomonas aeruginosa* is a Gram-negative opportunistic pathogen that causes severe infections in humans, in particular in cystic fibrosis patients (1). Aminoglycoside antimicrobials, such as tobramycin, gentamicin, neomycin and amikacin, are vital for treating *P. aeruginosa* infections by targeting the 30S ribosome and inhibiting protein synthesis to achieve bactericidal effect (2,3). *Pseudomonas aeruginosa* has become multidrug-resistant to widely used antibiotics by developing various antibiotic resistance mechanisms, including biofilm formation, persister formation, target modification, enzymatic drug modification, multidrug efflux systems and low membrane permeability (2,4).

*Pseudomonas aeruginosa* can rapidly adjust its metabolism for acclimating to the changes in the host environment, owing to the large number of regulatory proteins encoded by its genome (5,6). Transcription factors (TFs) sense the environmental stimulants and alter the expression of genes whose products are involved in drug resistance, metabolic pathways, pathogenicity of bacteria and so on. For example, RpiR family members usually control the expression of enzymes in different metabolic pathways (7–12), and MarR family numbers regulate the antibiotic resistance of bacteria (13–15).

Previous studies showed that certain metabolic stimulants, such as glucose, mannitol, fructose and pyruvate, can increase the susceptibility of *Escherichia coli* persisters to aminoglycoside antibiotics, and fructose also can sensitize *Staphylococcus aureus* persisters to aminoglycoside antibiotic killing (16). For *P. aeruginosa*, fumarate can boost the antimicrobial activity of tobramycin, and in contrast, glyoxylate can promote bacterial tolerance to tobramycin (17). Moreover, inactivation of the glycolytic enzyme, triosephosphate isomerase (*tpiA*), significantly reduced the bacterial resistance to aminoglycoside antibiotics (18). These previous findings well demonstrated that bacterial carbon flux is crucial for its tolerance to aminoglycoside antibiotics. However, the specific regulatory elements in *P. aeruginosa* that respond to these central metabolites and switch the antimicrobial susceptible states (tolerant or susceptible) remain elusive.

In this study, we screened a *P. aeruginosa* TF inactivation library generated by site-specific transposon-assisted genome engineering (STAGE) technology (19), and identified that a mutant carrying the inactivation of the PA5438 (*rccR*) gene, a member of the RpiR TF family, was significantly enriched under tobramycin selection. Transcriptomic analyses indicated that RccR can alter the bacterial carbon flux via regulating the expression of metabolic enzymes in pyruvate synthesis (*aceE* and *aceF*) and glyoxylate shunt pathway (*aceA* and *glcB*), and consequently, affecting the tolerance to aminoglycoside antibiotics. Moreover, through biochemical approaches, we confirmed that RccR responds to a metabolic intermediate of the Entner–Doudoroff pathway, 2-keto-3-deoxy-6-phosphogluconate (KDPG), for its regulatory role. To further understand the molecular mechanism of KDPG responding by RccR, we solved the structure of the RccR/KDPG complex and explored the functions of key residues that interact with KDPG. Collectively, these findings shed light on the molecular mechanism of RccR-mediated metabolic regulation and provided a plausible model for the development of aminoglycoside tolerance in *P. aeruginosa*.

## Materials and methods

### Bacterial strains, plasmids, primers and growth conditions

The strains and plasmids used in this study are listed in Supplementary Table S2. The primers used in this study are listed in Supplementary Table S3. The *E. coli* strains and *P. aeruginosa* strains were grown at 37°C in Luria–Bertani broth (LB). Antibiotics were added at the following concentrations: 15 μg/ml tetracycline, 50 μg/ml carbenicillin and 50 μg/ml kanamycin for *E. coli* strains; 100 μg/ml tetracycline and 150 μg/ml carbenicillin for *P. aeruginosa* strains.

### Construction of bacterial strains and plasmids

The pACRISPR/pCasPA system was used to construct the *rccR*-deletion strain in PAO1 as previously described (20). The pACRISPR-NN1-*rccR* plasmid was constructed to contain a spacer targeting the *rccR* gene and a repair template consisting of ∼500-bp flanking sequences at the upstream and the downstream of the *rccR* gene. The pACRISPR-NN1-*rccR* was then electroporated into the PAO1 wild-type electrocompetent cells containing pCasPA. Deletion of *rccR* was validated by polymerase chain reaction (PCR) and Sanger sequencing. Plasmids were then cured in successfully mutated cells. The *aceE*-deletion strain was similarly constructed.

To complement the *rccR* mutation, the pACRISPR-NN2-*rccR* plasmid was constructed to contain a spacer targeting the deletion junction and a repair template consisting of the complete *rccR* gene with a synonymous mutation, and ∼500-bp flanking DNA sequences at the upstream and the downstream of the *rccR* gene. The pACRISPR-NN2-*rccR* plasmid was electroporated into the *rccR*-deletion electrocompetent cells containing pCasPA. Complementation of *rccR* was validated by PCR and Sanger sequencing. Plasmids were then cured in successfully complemented cells. To introduce certain residue substitution in RccR, mutations were created on the pACRISPR-NN2-*rccR* plasmid by circular polymerase extension cloning (CPEC) (21) using 2× *Taq* Master Mix (GenScript). Complementation strains of certain RccR mutants were similarly created.

For expression and purification of RccR, the coding sequence of *rccR* gene from *P. aeruginosa* PAO1 was inserted into a pET28a backbone with a C-terminal His_6_ tag using Hieff Clone^®^ Universal One Step Cloning Kit (Yeasen). Mutations of *rccR* gene were then introduced into pET28a-RccR-his via CPEC. The constructed plasmids were extracted by using TIANprep Mini Plasmid Kit (TIANGEN).

For overexpressing the *aceA* gene or *glcB* gene in PAO1 wild-type strain, the coding sequences of *aceA* gene and *glcB* gene were inserted into a pAK1900 backbone with *rpsL* promoter of PAO1 strain, respectively. Then, the pAK1900-*rpsl-aceA* and pAK1900-*rpsl*-*glcB* plasmids were electroporated into the PAO1 electrocompetent cells.

### Protein expression and purification

The pET28a-RccR-his plasmid was transformed into *E. coli* BL21(DE3), and the cells were grown in LB with 50 μg/ml kanamycin at 37°C until the optical density at 600 nm (OD_600_) reached 0.6. The isopropyl-β-d-thiogalactopyranoside was supplemented at a final concentration of 0.25 mM to induce the protein expression overnight at 16°C. Cells were harvested by centrifugation and the pellet was resuspended in buffer A (10 mM Tris–HCl, pH 7.5, 1 M NaCl and 1 mM DTT), and then the suspension was disrupted by sonication and clarified by centrifugation. The supernatant was filtered and then loaded onto a 5-ml HisTrap Ni-NTA column (Cytiva). The column was washed with buffer A containing 62.5 mM imidazole for unbound proteins, and the His-tagged RccR protein was eluted with buffer A containing 500 mM imidazole. The eluted protein was concentrated to 2 ml and loaded onto a HiLoad 16/600 Superdex 200pg column (Cytiva) for further elution with buffer A. The purified protein was concentrated for subsequent experiments.

### Protein crystallization, data collection and structure determination

The RccR/KDPG complex was crystallized at 16°C by using the sitting-drop vapor-diffusion method. The RccR protein (6 mg/ml in 400 mM NaCl, 10 mM Tris–HCl, pH 7.5) was incubated with KDPG (the molar ratio of RccR and KDPG was ∼1:10) on ice for 30 min. One microliter of RccR/KDPG complex solution was mixed with an equal volume of reservoir solution containing 0.15 M dl-malic acid (pH 7.0), 0.1 M imidazole (pH 7.0) and 22% polyethylene glycol monomethyl ether 550 (v/v). The crystals were protected in liquid nitrogen before data collection.

The data were collected at BL18U1 beamline of the Shanghai Synchrotron Radiation Facility and processed by HKL3000 (22). The phase of the RccR/KDPG complex structure was determined by Phaser (23) from CCP4i (24) using the structure of *Vibrio vulnificus* NanR (PDB code: 4IVN) (25) as the search model. The model was built by Autobuild from PHENIX (26). The model of RccR/KDPG was refined using Refmac5 from CCP4i and further improved manually by Coot (27). The final structure figures were prepared by Pymol (http://www.pymol.org).

### Antibiotic screening assay of *P. aeruginosa* mutant library

The antibiotic screening assay of *P. aeruginosa* TF mutant library was performed as previously described (19). The TF mutation inactivation library of *P. aeruginosa* was constructed using the CRISPR/Cas12k transposition system (19). A glycerol stock of *P. aeruginosa* TF mutant library was thawed and mixed on ice. Then, 100 μl of cells were diluted into 100 ml of LB medium with or without 3 μg/ml tobramycin. The culture was shaken at 37°C overnight and harvested by centrifugation. The genomic DNA was extracted by the Ezup column bacteria genomic DNA purification kit (Sangon, Shanghai) and then subjected to NGS (HaploX Genomics Center, Jiangxi).

### Isothermal titration calorimetry

The association constants were determined by the MicroCal ITC200 system (Malvern). The RccR wild-type protein, RccR mutant proteins and the KDPG were prepared in the same buffer containing 10 mM Tris–HCl (pH 7.5) and 350 mM NaCl. The proteins and the KDPG were diluted to a final concentration of 60 and 600 μM, respectively. The KDPG solutions in the syringe were slowly titrated into the reaction cell containing the protein solutions. The whole process of the assay was carried out at 25°C with a stirring speed of 750 rpm. The ligand solutions were injected 20 times with 120 s intervals between two injections. The raw data were analyzed with the Origin7 software package (Malvern).

### Electrophoretic mobility shift assay

The DNA substrates for electrophoretic mobility shift assay (EMSA) were prepared by PCR amplification using FAM-labeled primers from the *P. aeruginosa* PAO1 genome and then gel purification by a SPARKeasy Gel/PCR Purification Kit (Shandong Sparkjade Biotechnology Co., Ltd). The labeled DNA fragments (10 nM) were incubated with different concentrations of purified protein on ice for 30 min in the reaction buffer (20 mM Tris–HCl, pH 8.5, 150 mM KCl, 5 mM MgCl_2_, 1 mM EDTA, 1 mM DTT, 0.8% Tween 20 and 2.5% glycerol). The mixtures were added with 5× bromophenol blue loading buffer and then subjected to 6% native polyacrylamide gel electrophoresis in 0.5× TBE buffer at 120 V for 30 min. Images were acquired by the GelDoc System (Bio-Rad).

The labeled DNA fragments (10 nM), a certain concentration of purified RccR protein and different concentrations of KDPG were incubated on ice for 30 min in the reaction buffer and then separated on 6% native polyacrylamide gels using the same protocol.

### Growth curve assays

Two microliters of overnight cultures of different *P. aeruginosa* PAO1 strains (∼OD_600_= 1.4) were diluted into 200 μl of fresh LB medium or MOPS minimal medium and transferred to a BioScreen micro-well plate. The plate was shaken continuously at 37°C in the automated microbe growth curve analysis system BioScreen C (OY Growth Curves Ltd, Finland) and the OD_600_ was measured every 1 h. The MOPS minimal medium was prepared as previously described (28), and supplemented with 20 mM glucose, 20 mM glycerol and 20 mM acetate as the sole carbon sources, respectively. All experiments were performed in triplicate.

### Spotting assay

Overnight cultures of different *P. aeruginosa* PAO1 strains were subjected to eight consecutive 10-fold dilutions in LB medium. The diluted bacteria were spotted on the LB agar plates supplemented with or without antibiotics. The plates were incubated overnight at 37°C.

### RNA extraction and transcriptome sequencing

*Pseudomonas aeruginosa* PAO1 wild-type and *rccR*-deletion mutant strains were cultured in LB medium at 37°C and harvested at the exponential phase and stationary phase, respectively. The total RNAs were extracted using the MiniBEST Universal RNA Extraction Kit (Takara) and used as templates for reverse transcription to complementary DNA (cDNA) using the TransScript^®^ One-Step gDNA Removal and cDNA Synthesis SuperMix (TransGen Biotech Co.) according to the manufacturer’s instructions. The cDNA products were subjected to transcriptome sequencing by Majorbio (Shanghai).

### Quantitative real-time PCR

Quantitative real-time PCR (qRT-PCR) was carried out in the CFX96™ Touch Real-Time PCR Detection System (Bio-Rad) by using ChamQ Universal SYBR qPCR Master Mix (Vazyme). Gene expression level was normalized to the internal reference, the *gyrB* gene of *P. aeruginosa*. Three biological replicates with three technical replicates were performed for all experiments.

## Results

### *rccR* is associated with aminoglycoside antibiotic tolerance

**Figure 1.**
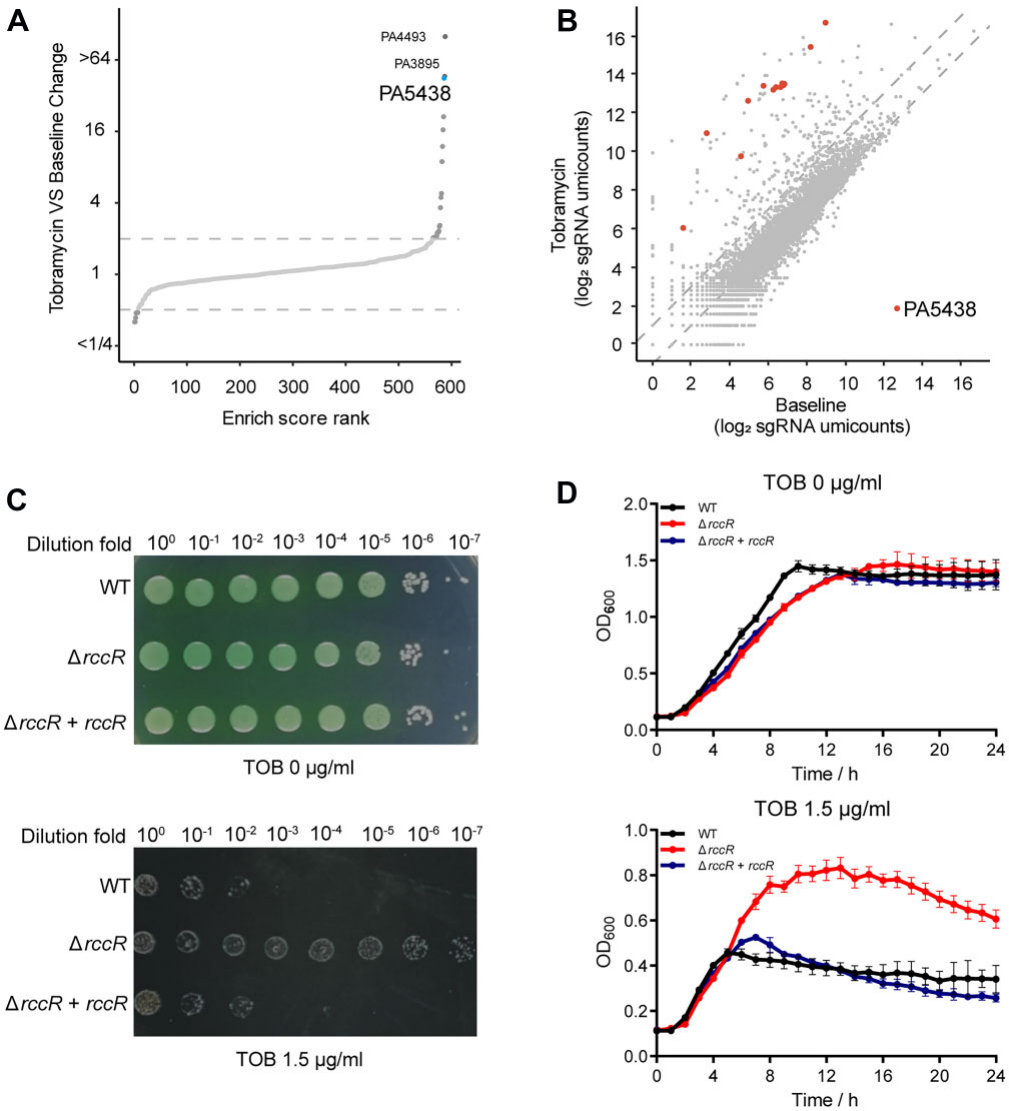
The *rccR*-deletion mutant strain is tolerant to tobramycin. (**A**) Enrich scores of each gene are ranked and plotted. PA5438 (*rccR*) is enriched after tobramycin treatment and marked in blue color. (**B**) Scatterplot shows the insertion counts of all target sites in tobramycin-treated and control samples. The sites belonging to PA5438 are marked in red color. (**C**) Spotting assay for the *rccR*-deletion mutant. The strains are grown in LB agar plates in the presence or absence of tobramycin. (**D**) Growth curve assay for the *rccR*-deletion mutant. The strains are grown in LB medium in the presence or absence of tobramycin. Data are represented as mean ± standard deviation (SD) (*n* = 3).

To explore the TFs associated with aminoglycoside antibiotic tolerance in *P*. *aeruginosa*, we treated the TF mutant library that was constructed by CRISPR/Cas12k-guided STAGE technology with tobramycin (3 μg/ml) as previously described (19). The TF library was recovered from frozen cultures and cultured in fresh LB medium or LB containing tobramycin (Supplementary Figure S1A). The drug-tolerant mutants survived and were enriched during this process, whereas the antibiotic-sensitive mutants were gradually eliminated. We performed next-generation sequencing (NGS) analysis on the samples with and without tobramycin treatment and compared the guide reads of each gene in the tobramycin-treated sample with those in the untreated control sample. We found that disruption of *rccR* substantially increased *P*. *aeruginosa* tolerance to tobramycin (Figure 1A and Supplementary Figure S1B), and the guides targeting PA5438 (*rccR*) were enriched (Figure 1B), indicating that the disruption of *rccR* indeed contributes to bacterial growth in the presence of tobramycin.

To further validate the *rccR*-mediated aminoglycoside antibiotic tolerance, we constructed the *rccR*-deletion mutant and *rccR*-complementation strains using the CRISPR/Cas9 genome editing system in *P*. *aeruginosa* (20). As shown in Figure 1C and D, deletion of *rccR* significantly increased bacterial tolerance to tobramycin, and this phenotype can be restored by introducing a wild-type copy of *rccR* into the *rccR*-deletion mutant strain. Moreover, consistent results were observed with the treatment of two additional aminoglycoside antibiotics, gentamicin and amikacin (Supplementary Figure S1C and D). However, we did not observe the growth differences between the wild-type strain and *rccR*-deletion mutant in the treatment with other classes of antibiotics (Supplementary Figure S2A–F), suggesting that the *rccR*-deletion mutant exclusively increases bacterial tolerance to aminoglycoside antibiotic in *P*. *aeruginosa*.

### RccR regulates the pyruvate catabolism and glyoxylate shunt pathway

**Figure 2.**
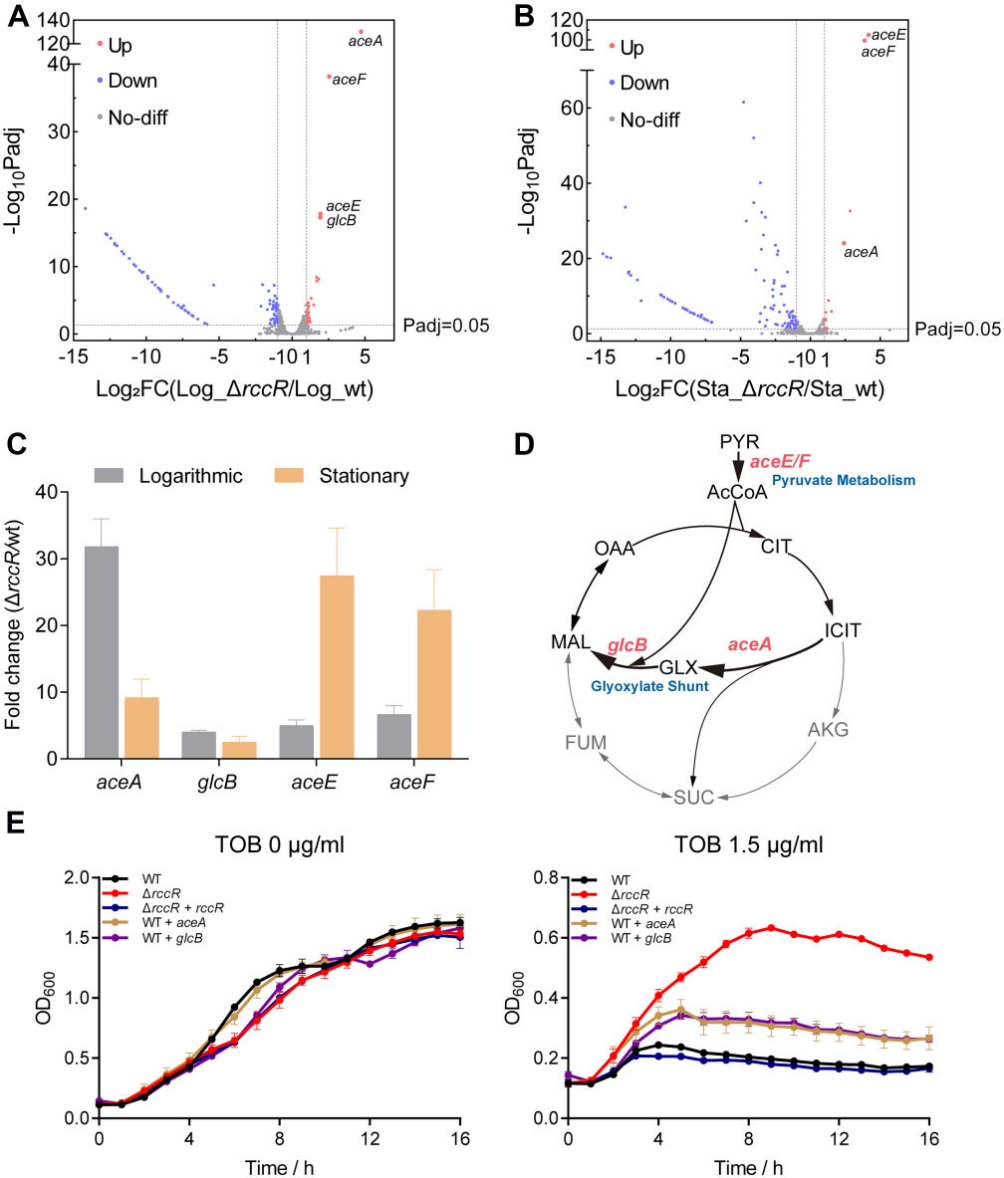
RccR is a regulator of pyruvate metabolism and glyoxylate shunt pathway. Volcano plots for significance difference analysis of gene expression between PAO1 wild-type and *rccR*-deletion mutants in the logarithmic phase (**A**) and stationary phase (**B**). Red circles indicate upregulated expression, blue circles indicate downregulated expression and gray circles indicate no significant difference. (**C**) The relative mRNA transcription levels of *aceE*, *aceF*, *aceA* and *glcB* of the wild-type PAO1 and the *rccR*-deletion mutant in the logarithmic phase and stationary phase. The *gyrB* gene of PAO1 is used as the reference gene. Data are represented as mean ± SD (*n* = 3). (**D**) The genes in pyruvate metabolism and glyoxylate shunt pathway are regulated by RccR. (**E**) Growth curve assay of the PAO1 strains overexpressing *aceA* or *glcB* gene. The strains are grown in LB medium in the presence or absence of tobramycin. Data are represented as mean ± SD (*n* = 3).

To investigate the regulatory roles of RccR, we performed the transcriptome analysis of the PAO1 wild-type and *rccR*-deletion mutant strains. As shown in Figure 2A and B, Supplementary Figure S3 and Supplementary Table S4, the expression of most of the identified genes was significantly downregulated when *rccR* was deleted, whereas the expression of *aceE*, *aceF*, *aceA* and *glcB* was substantially increased (Figure 2A and B), suggesting that RccR was a transcriptional repressor of these genes. We further measured the mRNA transcription levels of *aceE*, *aceF*, *aceA* and *glcB* by qRT-PCR, showing a result consistent with the RNA sequencing (RNA-seq) result (Figure 2C), thereby confirming the regulatory role of RccR in pyruvate catabolism (*aceE* and *aceF*) and glyoxylate shunt pathway (*aceA* and *glcB*) (Figure 2D).

A previous study confirmed that the tricarboxylic acid (TCA) cycle and cellular respiratory activity are both essential for aminoglycoside antibiotics lethality (17). Therefore, we hypothesized that the tolerance of *rccR*-deletion strain to aminoglycoside antibiotic may be related to the activation of the glyoxylate shunt pathway (Figure 2D), which disrupted downstream TCA cycle activity. To verify this hypothesis, we overexpressed *aceA* gene and *glcB* gene into the PAO1 wild-type strain by a pAK1900 plasmid (*rpsl* promoter), respectively, and measured the growth curve in the presence or absence of aminoglycoside antibiotics. As shown in Figure 2E and Supplementary Figure S4A and B, overexpression of *aceA* gene or *glcB* gene in wild-type strain enhanced the tolerance to aminoglycoside antibiotics compared to wild-type and complementation strains, suggesting that increasing the expression of enzymes in the glyoxylate shunt pathway may lead to aminoglycoside antibiotic tolerance.

### RccR is a KDPG-responsive regulator

**Figure 3.**
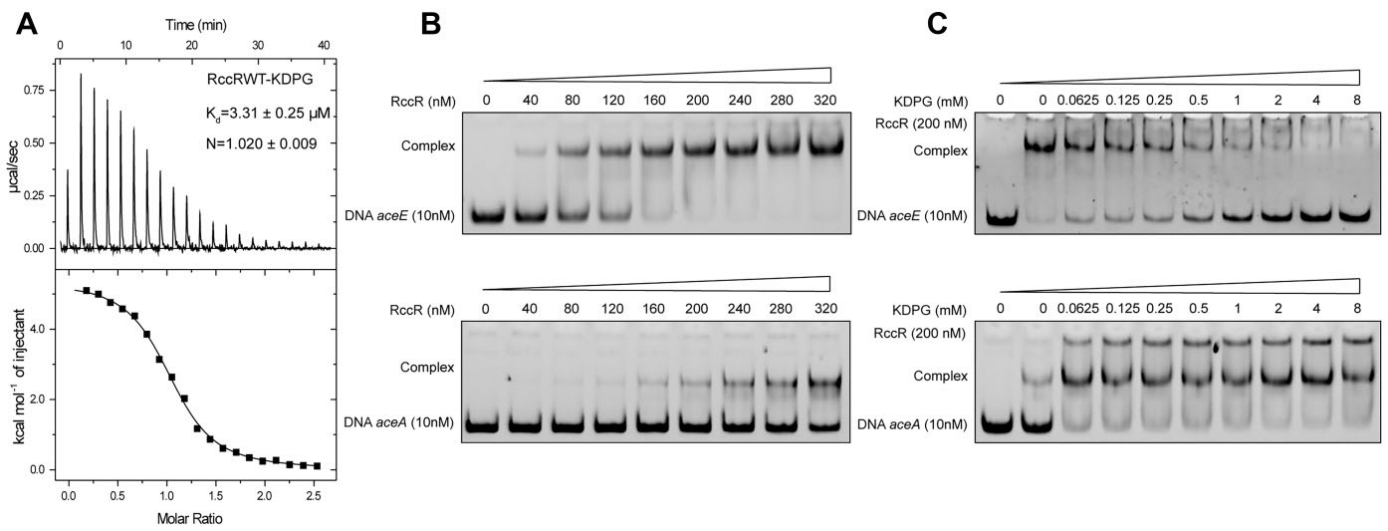
Assessment of the effect of KDPG on RccR. (**A**) ITC assay for the binding between RccR and KDPG. *K*_d_, dissociation constant; *N*, number of binding sites per RccR. EMSA analysis of the interaction between RccR and its operator DNA in the absence (**B**) or presence (**C**) of 0.0625, 0.125, 0.25, 0.5, 1, 2, 4 and 8 mM KDPG.

A previous study demonstrated that KDPG is the signal molecule for the RccR homolog in *P. fluorescens* (8). To explore whether KDPG is also an effector of RccR in *P. aeruginosa*, we performed the isothermal titration calorimetry (ITC) to determine the binding affinity between RccR and different carbon metabolites. The ITC data showed that except for KDPG, no response was observed for citrate, succinate or malonate when they were used for the titration (Figure 3A and Supplementary Figure S5A–C). RccR bound to KDPG with a dissociation constant (*K*_d_) value of 3.31 μM and nearly 1:1 binding stoichiometry (Figure 3A), indicating that one RccR molecule binds one molecule of KDPG.

To investigate the impact of KDPG binding on RccR-mediated regulation, we performed the EMSA between RccR and its operator DNA in the presence or absence of KDPG. EMSA showed that RccR can effectively bind to the promoter DNA, and the binding affinity between RccR and the promoter DNA of *aceE* was stronger than that of *aceA* or *glcB* (Figure 3B and Supplementary Figure S6A). Intriguingly, the addition of KDPG significantly relieved the retardation of *aceE* promoter DNA migration, suggesting that KDPG interfered the binding of RccR to the *aceE* promoter DNA. In contrast, KDPG addition strengthened the binding affinity of RccR to both promoters of *aceA* and *glcB* (Figure 3C and Supplementary Figure S6A). These results showed that RccR is a KDPG-sensing regulator with two distinct regulatory effects upon KDPG binding: one is to relieve the repression of pyruvate metabolism and the other is to increase the repression of the glyoxylate shunt pathway (Supplementary Figure S6B).

### Structural characterizations of the RccR/KDPG complex

**Figure 4.**
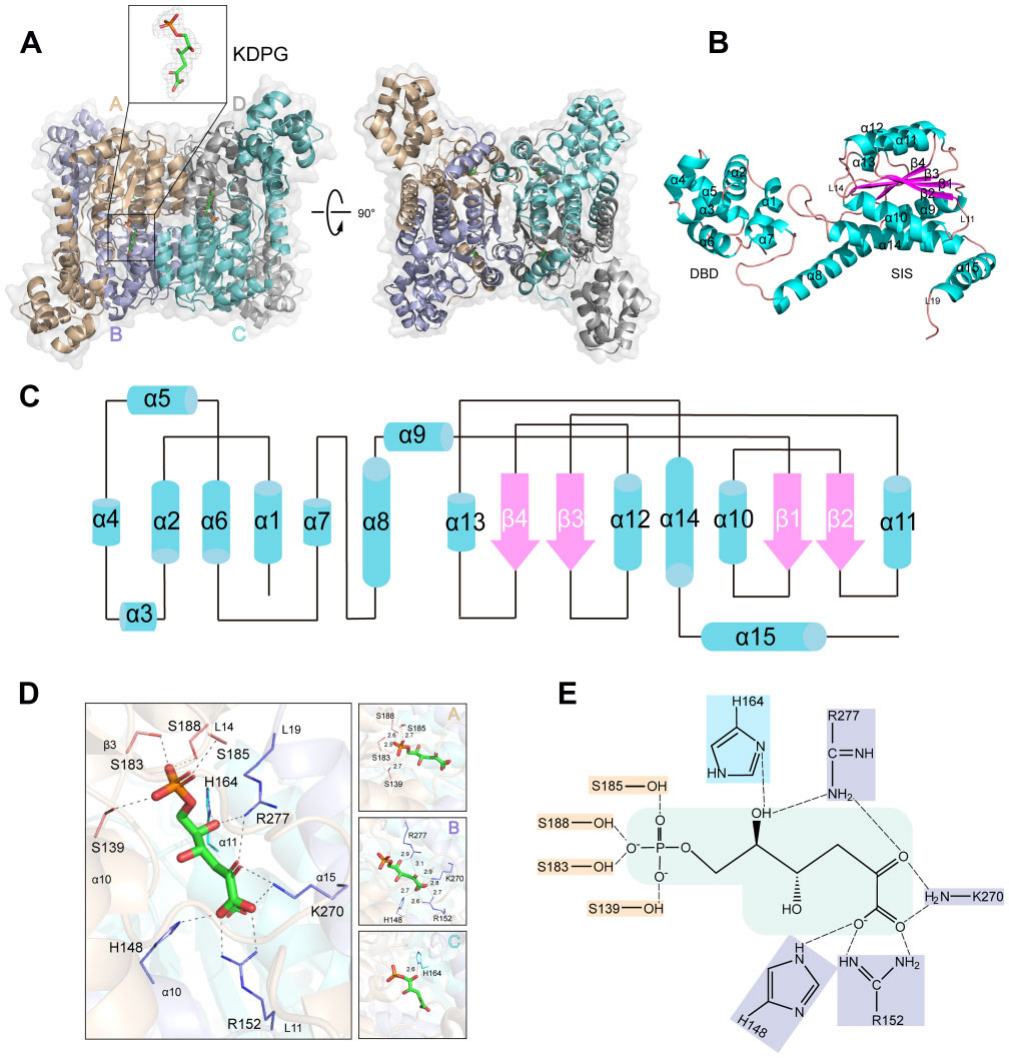
Structural characterizations of the RccR/KDPG complex. (**A**) The overall structure of RccR/KDPG complex in tetrameric form and the detailed view of the electron density of KDPG. The monomers (A–D) are marked with wheat, light blue, aquamarine and gray, respectively. KDPG is colored in green. (**B**) Cartoon of RccR in its monomeric structure. α-Helices, β-sheets and loops are colored in cyan, magenta and pink, respectively. (**C**) Schematic representation of the topology of RccR based on the tertiary structure of RccR/KDPG. (**D**, **E**) The detailed direct hydrogen-bond interactions between RccR and KDPG. Dashed lines represent direct hydrogen bonds. Residues located in the same monomer are marked with the same color.

To elucidate the detailed KDPG-sensing and regulatory mechanism of RccR, we attempted to determine the crystal structures of apo-RccR and the RccR/KDPG complex. We screened hundreds of crystallization conditions and obtained the crystals of the RccR/KDPG complex. The complex was crystallized with the space group *I222*, and the crystal structure was refined to 1.9 Å resolution (Supplementary Table S1). Structural analysis revealed that the RccR/KDPG complex contains four monomers (A–D) with each monomer consisting of two domains (Figure 4A and B): an N-terminal DNA-binding domain (DBD) and a C-terminal sugar isomerase domain (SIS). The DBD comprises seven α-helices and the SIS forms an α/β structure, which consists of four-stranded parallel β-sheets and eight α-helices (Figure 4B and C). The last α-helix is absent in the SIS due to the poor electron density. Each monomer in this complex structure harbors a well-defined signal molecule KDPG (Figure 4A), which resides in the central pocket surrounded by three individual SISs.

We failed to obtain the crystal of apo-RccR, likely because of the high flexibility of RccR in the absence of KDPG. We thereby predicted the structure of apo-RccR using ColabFold (29–31). As shown in Supplementary Figure S7A, compared to the structure of the RccR/KDPG complex, apo-RccR displays an open conformation with the two domains relatively scattered. We overlapped SISs in one monomer of apo-RccR with that of the RccR/KDPG complex and observed that all the DBDs were separate and significant conformational changes occurred in SISs of the other three monomers (Supplementary Figure S7B). Taken together, these results indicated that KDPG is a specific signal molecule that stabilizes the conformation of RccR protein, binding of which may trigger a conformational change to RccR.

### KDPG recognition mechanism by RccR

Extensive interactions between RccR and KDPG were revealed by a close inspection of the ligand-binding pocket of the RccR/KDPG complex structure. In total, nine residues (A.S139, B.H148, B.R152, C.H164, A.S183, A.S185, A.S188, B.K270 and B.R277), involving three different monomers (A–C) of the tetrameric protein, engage in direct hydrogen bonds with KDPG (Figure 4D). Specifically, KDPG interacts with RccR via the sites formed by α10 helix (S139), β3 sheet (S183) and L14 loop (S185 and S188) of monomer A, α10 helix (H148), L11 loop (R152), α15 helix (K270) and L19 loop (R277) of monomer B, and α11 helix (H164) of monomer C (Figure 4D). The side-chain hydroxyl groups of four serine residues (A.S139, A.S183, A.S185, A.S188) play a main role in recognizing the phosphate group and forming hydrogen bonds with the phosphate oxygen atoms of KDPG (Figure 4D and E). The side-chain amines of B.H148, B.R152 and B.K270 provide hydrogen-bond interactions with the carboxyl oxygen atom at position C1 of KDPG, and the carbonyl group at position C2 also forms hydrogen bonds with the side-chain amines of B.K270 and B.R277. Moreover, the hydroxyl group at position C5 of KDPG forms hydrogen bonds with the side-chain imidazole nitrogen atom of C.H164 and the side-chain amine of B.R277 (Figure 4D and E).

### KDPG binding by RccR is critical for RccR-mediated regulation

**Figure 5.**
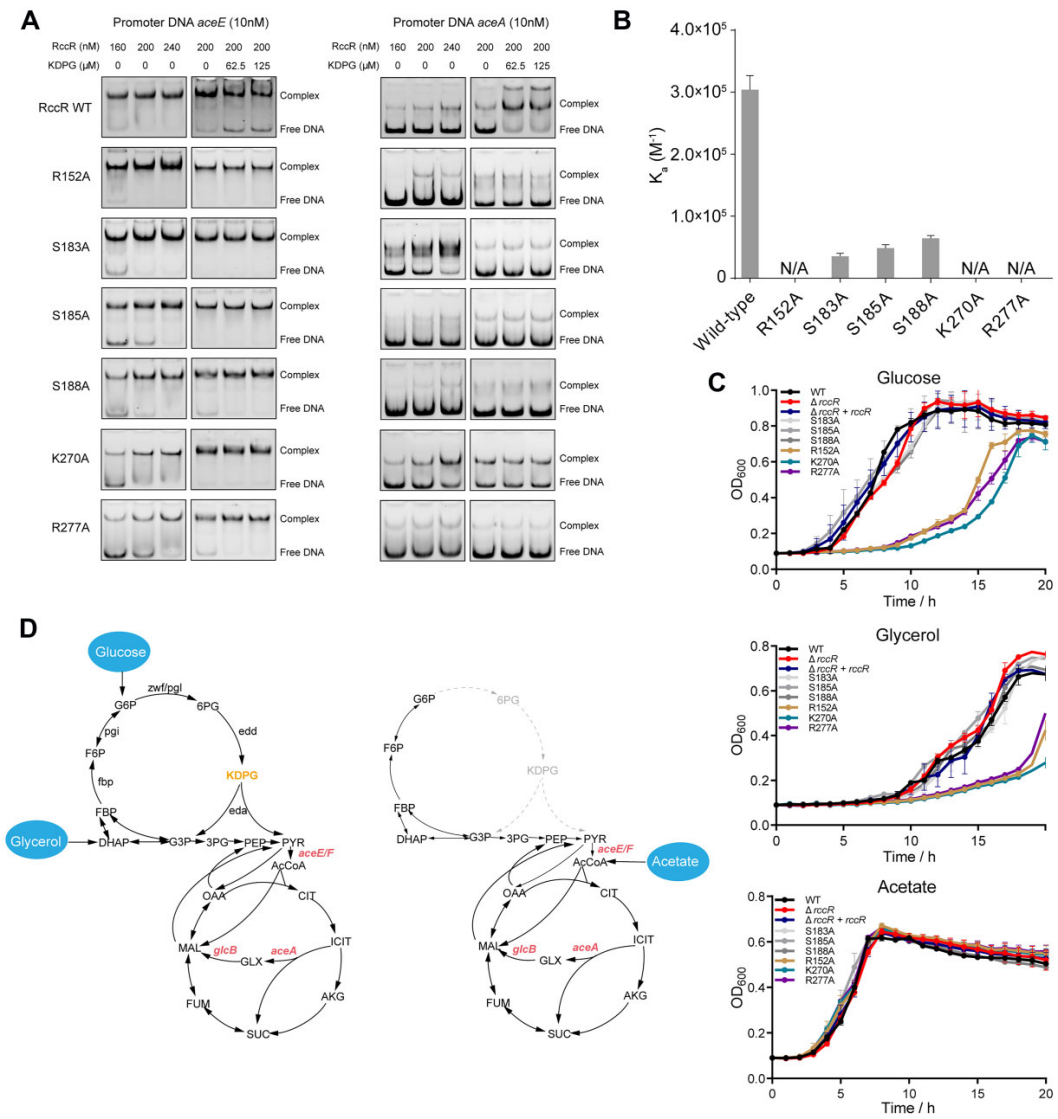
KDPG recognition by RccR is crucial for the regulation activity. (**A**) EMSA analysis of the binding abilities of RccR single mutant proteins to different DNAs in the absence or presence of increasing amounts of KDPG. The gel electrophoresis images of RccR WT protein are from Figure 3B and C. WT, wild type. (**B**) ITC assays for the binding affinities between KDPG and different RccR single mutant proteins. *K*_a_, association constant; N/A, no detectable binding by ITC. (**C**) Growth curves of PAO1 WT and *rccR* mutant strains in MOPS minimal medium supplemented with glucose, glycerol or acetate as the sole carbon source. WT, wild type. Data are represented as mean ± SD (*n* = 3). (**D**) Carbon metabolism in *P. aeruginosa* with glucose, glycerol or acetate as the sole carbon source.

To verify the roles of these residues in KDPG binding, we mutated these residues (S139, H148, R152, H164, S183, S185, S188, K270 and R277) to alanine (A), respectively. We purified the mutant proteins and performed EMSA to assess the promoter DNA binding activity of mutant proteins. Compared with wild-type RccR, R152A, S183A, S185A, S188A, K270A and R277A mutant proteins exhibited similar binding activities to the target DNAs (Figure 5A), whereas mutation of S139, H148 or H164 to alanine abolished the binding activity of RccR to the same target DNAs (Supplementary Figure S8). Furthermore, in contrast to the wild-type RccR, the R152A, S183A, S185A, S188A, K270A and R277A mutant proteins were almost unresponsive to KDPG treatment (Figure 5A), indicating that the mutation of these residues impaired the interaction between RccR and KDPG.

Next, we employed ITC to quantitatively examine the binding affinities between the mutant proteins (R152A, S183A, S185A, S188A, K270A and R277A) and KDPG. As shown in Figure 5B and Supplementary Figure S9, compared with wild-type RccR, the S183A, S185A and S188A mutant proteins showed significantly weakened binding affinities to KDPG with *K*_d_ of 28.66, 20.97 and 15.69 μM, respectively (Supplementary Figure S9A–C); the R152A, K270A and R277A mutant proteins exhibited undetectable binding activities to KDPG (Supplementary Figure S9D–F), suggesting that these three residues are essential for KDPG recognition.

Given the vital role of RccR in regulating pyruvate catabolism and glyoxylate shunt pathway, we assessed the growth of these mutant strains in MOPS minimal medium supplemented with different sole carbon sources. The R152A, S183A, S185A, S188A, K270A and R277A mutations were individually introduced into *P. aeruginosa* by using CRISPR/Cas9-based genome editing strategy (20), and then the growth curves of these mutant strains were measured in different culture conditions. Interestingly, three mutant strains (R152A, K270A and R277A) showed impaired growth in MOPS medium supplemented with glucose or glycerol, but not acetate, as the sole carbon sources (Figure 5C), whereas all the other strains displayed similar growth irrespective of the carbon source (Figure 5C).

Mutation of R152, K270 or R277 to alanine fully abolished KDPG binding by RccR (Figure 5B and Supplementary Figure S9D–F), which may permanently suppress the pyruvate catabolism pathway by constantly inhibiting the expression of *aceE/F*. Permanent suppression of pyruvate catabolism can result in attenuated acetyl-CoA production and downstream TCA cycle activity, and finally lead to growth impairment when glucose or glycerol is used as the carbon source (Figure 5C and D). However, acetate can be directly converted to acetyl-CoA and then fluxed to TCA cycle by bypassing the pyruvate catabolism pathway; thus, no growth inhibition was observed in the R152A, K270A and R277A mutants, in which pyruvate catabolism is constantly suppressed (Figure 5C and D). The role of the pyruvate catabolism pathway and *aceE/F* for bacterial growth when glucose or glycerol is used as the sole carbon source was further demonstrated as the PAO1 *aceE*-deletion strain grows slower in LB medium (Supplementary Figure S10A) and barely grows in MOPS medium supplemented with glucose compared with the PAO1 wild-type strain (Supplementary Figure S10B). Moreover, a similar growth phenotype was observed in *E*. *coli aceE*-deletion strain (32).

## Discussion

*Pseudomonas* *aeruginosa* is a Gram-negative opportunistic pathogen with metabolic versatility and the capacity to cause severe infections in humans (1,5). The superior metabolic plasticity of *P. aeruginosa* can be largely attributed to the sophisticated transcriptional regulatory networks (5,6), enabling its success in infection and antibiotic tolerance. Aminoglycoside is a class of commonly used antibiotics for treating *P. aeruginosa* infections (2,3). Metabolic perturbation by some central metabolites, such as fumarate and glyoxylate, was found to alter *P. aeruginosa* aminoglycoside antibiotic tolerance by adjusting TCA cycle activity (17). However, the linkage among transcriptional regulation, metabolic status and antibiotic tolerance in *P. aeruginosa* remains largely unclear.

Through genetic screening with the TF mutant library (19), we identified three TFs associated with aminoglycoside antibiotic tolerance, including a RoxS/RoxR two-component system regulator PA4493 (33), a RpiR family regulator PA5438 and an uncharacterized protein PA3895 (Figure 1A and Supplementary Figure S1B). The RpiR family regulators commonly act as transcriptional activators or repressors of sugar catabolism, such as maltose, glucose, lactose and galactose (7–10,12). Additionally, the homolog of PA5438 has been demonstrated to be a regulator of central carbon metabolism in *P*. *fluorescens* (8). We thereby chose PA5438 as a candidate regulator for central carbon metabolism and antibiotic tolerance for subsequent studies.

Transcriptome analysis combined with biochemical assays demonstrates the direct regulatory roles of RccR in pyruvate metabolism and glyoxylate shunt pathway. The enhancement of the glyoxylate shunt pathway and the resulting altered TCA cycle activity is likely a mechanism for the increased aminoglycoside antibiotic tolerance in the *rccR* mutant. In addition to the upregulation of *aceE*, *aceF*, *aceA* and *glcB* genes, many genes involving transport, metabolism, bacterial chemotaxis, quorum sensing and biofilm formation are found to be downregulated in the *rccR*-deletion mutant (Supplementary Figure S3 and Supplementary Table S4). Therefore, other mechanisms may also be associated with aminoglycoside antibiotic tolerance, which is further evidenced by the fact that overexpression of *aceA* or *glcB* cannot completely render the strain aminoglycoside antibiotic tolerance to the same level as that of the *rccR*-deletion mutant (Figure 2E and Supplementary Figure S4).

Taken together, we identified RccR as a TF that is associated with aminoglycoside antibiotic tolerance and regulates pyruvate metabolism and glyoxylate shunt pathway in *P*. *aeruginosa*, revealing the linkage among carbon metabolism, transcriptional regulation and drug tolerance (Supplementary Figure S11). Moreover, we determined the structure of the RccR/KDPG complex, elucidating the molecular mechanisms of KDPG sensing and RccR-mediated transcriptional regulation. This study provides insights into new antimicrobial strategy development against *P*. *aeruginosa* infections.

## Supplementary Material

gkad1201_supplemental_file

## Data Availability

The data underlying this article are available in the article and its supplementary information. The crystal structure of RccR/KDPG has been deposited in the RCSB Protein Data Bank (www.wwpdb.org) under the accession code 8K3B. The RNA-seq data have been deposited at Gene Expression Omnibus, and the accession number is GSE237535. The NGS data of screening the *P*. *aeruginosa* TF mutant library have been deposited at Sequence Read Archive, and the accession number is PRJNA995063.
