# Supplementary material for: A KDPG sensor RccR governs Pseudomonas aeruginosa carbon metabolism and aminoglycoside antibiotic tolerance
Source: Nucleic Acids Res. 2023 Dec 14;52(2):967–76. doi: 10.1093/nar/gkad1201 (PMC10810197; doi:10.1093/nar/gkad1201)
Supplement: gkad1201_supplemental_file [file gkad1201_supplemental_file.pdf]

Supplementary Information for

A KDPG sensor RccR governs *Pseudomonas aeruginosa* carbon metabolism and aminoglycoside antibiotic tolerance

Yujue Wang[1], Zhipeng Wang[1], Weizhong Chen[1], Ze-Hui Ren[2], Hui Gao[1], Jiani Dai[1], Guan-Zheng Luo[2], Zhaowei Wu[1,*], Quanjiang Ji[1,3,4*]

[1]School of Physical Science and Technology, ShanghaiTech University, Shanghai 201210, China.
[2]MOE Key Laboratory of Gene Function and Regulation, Guangdong Province Key Laboratory of Pharmaceutical Functional Genes, State Key Laboratory of Biocontrol, School of Life Sciences, Sun Yat-sen University, Guangzhou 510275, Guangdong, China.
[3]Gene Editing Center, School of Life Science and Technology, ShanghaiTech University, Shanghai 201210, China.
[4]Shanghai Clinical Research and Trial Center, Shanghai, 201210, China.

[*]Correspondence should be addressed to
Z.W. (wuzw1@shanghaitech.edu.cn);
Q.J. (quanjiangji@shanghaitech.edu.cn).

Table of Contents

Figure S1. Disruption of *rccR* is tolerant to aminoglycoside antibiotics.
Figure S2. Spotting assay of PAO1 WT, *rccR*-deletion mutant, and *rccR* complementation strains in the presence of different classes of antibiotics.
Figure S3. Grouping RccR-regulated genes according to their annotated function.
Figure S4. Growth curves of PAO1 strains overexpressing *aceA* or *glcB* in the presence of amikacin or gentamicin.
Figure S5. ITC assay of the binding affinities between RccR and citrate (A), succinate (B), and malonate (C).
Figure S6. The model of RccR regulation in primary carbon metabolism.
Figure S7. Structural characterizations of RccR.
Figure S8. The EMSA analysis of the interaction between S139A, H148A, and H164A single mutant RccR proteins and the promoter DNA.
Figure S9. ITC assay of the binding affinities between KDPG and single mutant RccR proteins.
Figure S10. The growth curves of PAO1 WT and *aceE*-deletion strains in LB medium and MOPS minimal medium supplemented with glucose.
Figure S11. The schematic illustration of aminoglycoside antibiotics tolerance, the E-D pathway, pyruvate catabolism, the glyoxylate shunt, and RccR transcriptional regulation.
Table S1. Data collection, phasing, and refinement statistics of the structures of RccR/KDPG

complex.

Table S2. Bacterial strains and plasmids used in this study.

Table S3. Primers used in this study.

Table S4. Transcriptome sequencing results in this study.

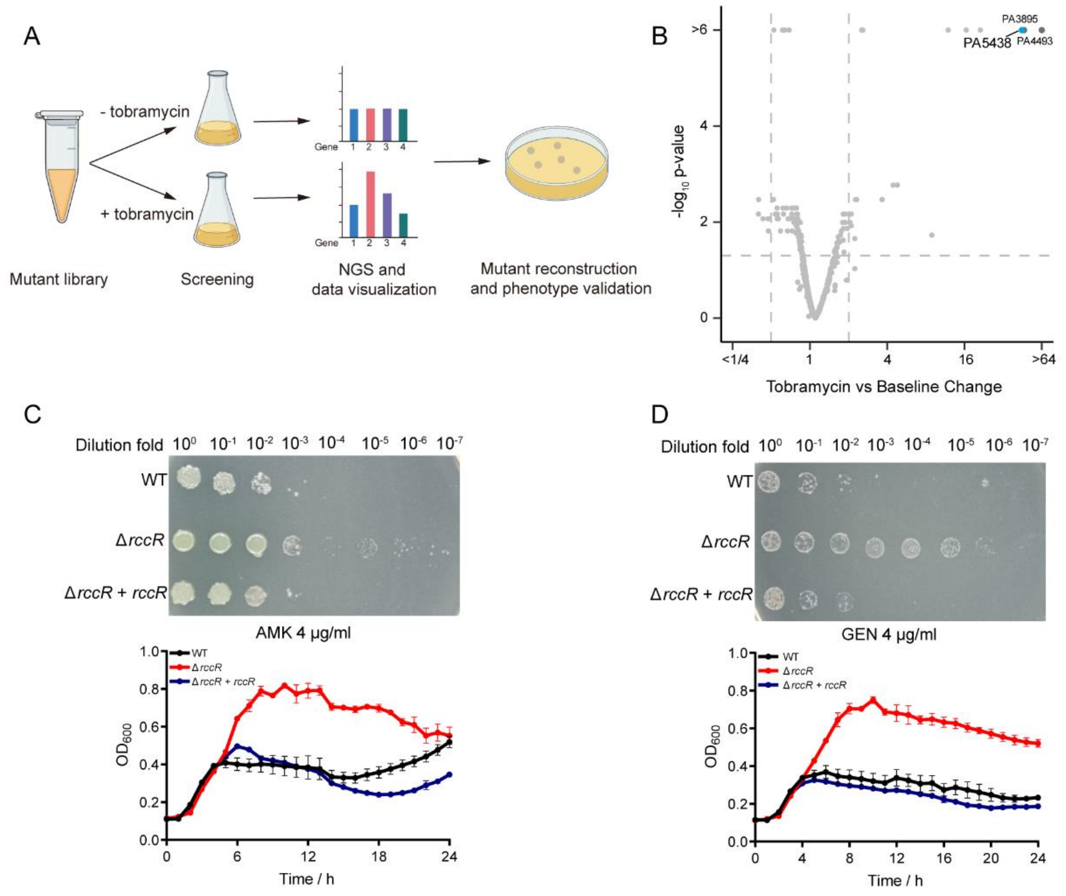

Figure S1. Disruption of *rccR* is tolerant to aminoglycoside antibiotics.

A. Scheme of the screening strategy using the *P*. *aeruginosa* TF mutant library.

B. Volcano plot shows PA5438 (*rccR*) gene is enriched after tobramycin treatment. The PA5438 gene is marked in blue color.

C and D. Spotting assay and growth curve assay of PAO1 WT, *rccR*-deletion mutant, and *rccR* complementation strains are treated with amikacin (C) or gentamicin (D). WT, wild-type; AMK, amikacin; GEN, gentamicin. The growth curve data are represented as mean ± SD (n = 3).

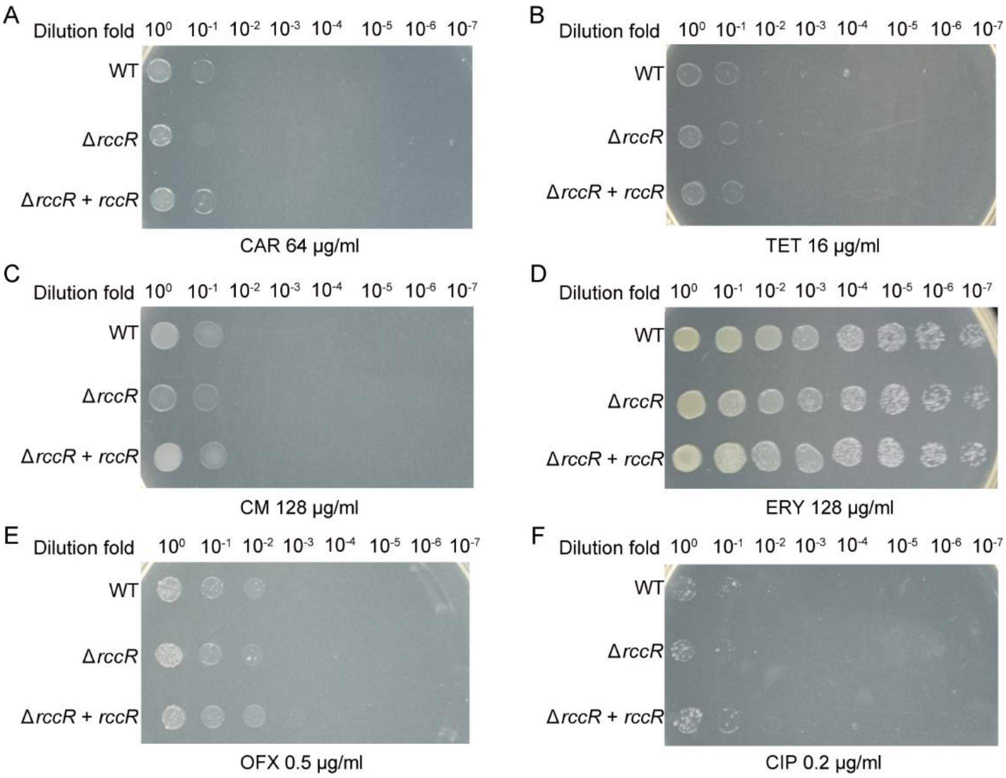

Figure S2. Spotting assay of PAO1 WT, *rccR*-deletion mutant, and *rccR* complementation strains in the presence of different classes of antibiotics. (A) β-lactam: CAR, carbenicillin; (B) Tetracycline: TET, tetracycline; (C) Chloramphenicol: CM, chloramphenicol; (D) Macrolides: ERY, erythromycin; (E-F) Quinolones: OFX, ofloxacin; CIP, ciprofloxacin. WT, wild-type.

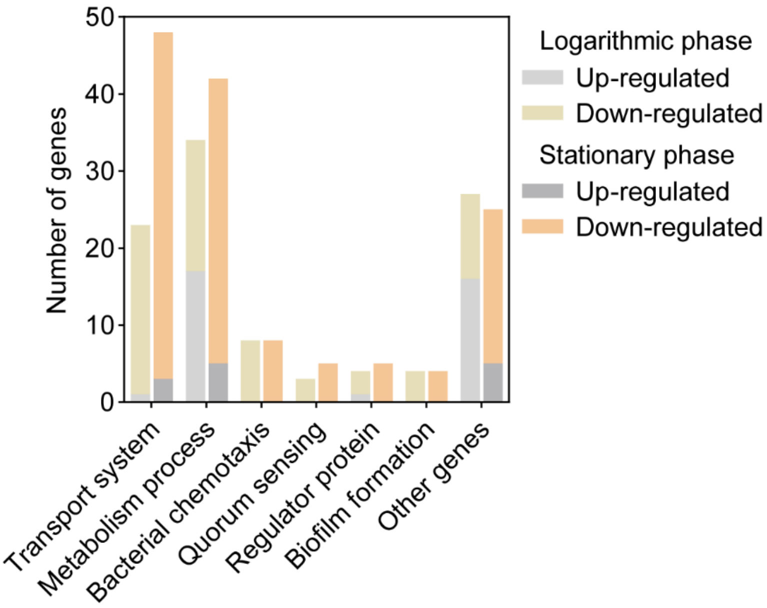

Figure S3. Grouping RccR-regulated genes according to their annotated function. Numbers of genes whose expressions are down-regulated or up-regulated in the *rccR*-deletion strain compared with the PAO1 wild-type strain in the logarithmic phase and stationary phase are shown.

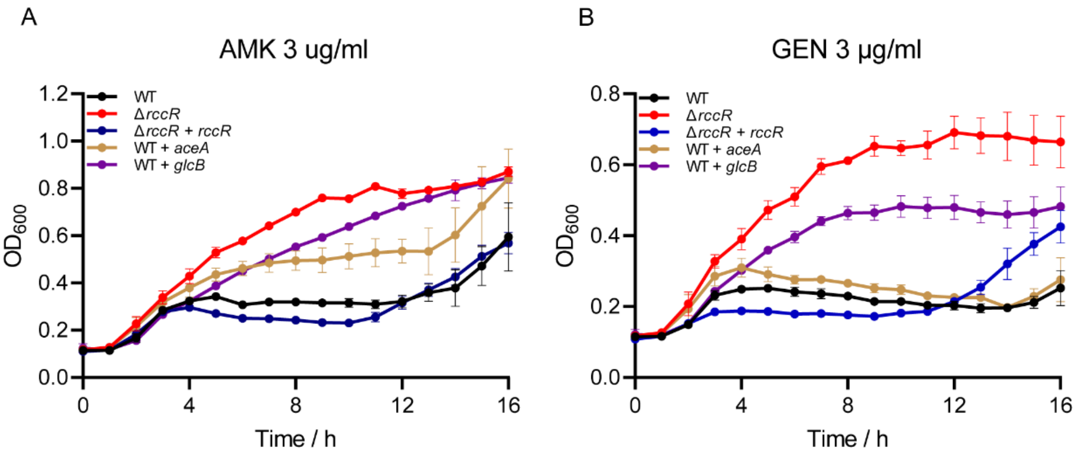

Figure S4. Growth curves of PAO1 strains overexpressing *aceA* or *glcB* in the presence of amikacin (A) or gentamicin (B). WT, wild-type; AMK, amikacin; GEN, gentamicin. Data are represented as mean ± SD (n = 3).

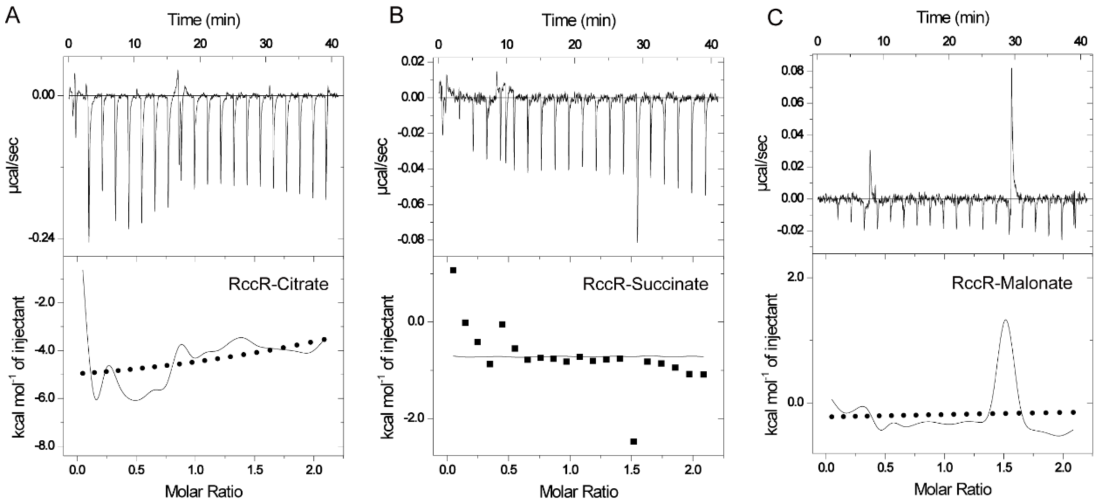

Figure S5. ITC assay of the binding affinities between RccR and citrate (A), succinate (B), and malonate (C).

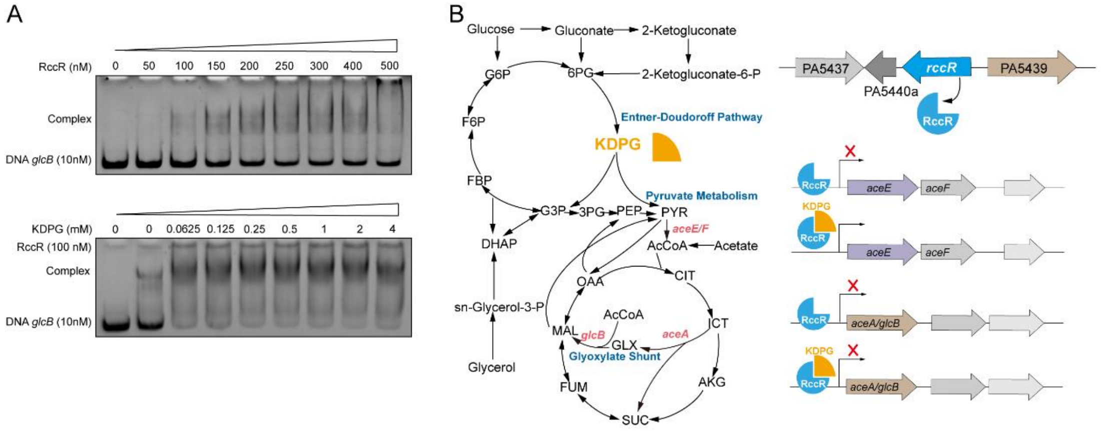

Figure S6. The model of RccR regulation in primary carbon metabolism.

A. The EMSA of the interaction between RccR and *glcB* promotor DNA in the absence or presence of 0.0625, 0.125, 0.25, 0.5, 1, 2, 4 mM KDPG.

B. Scheme of two completely different mechanisms of RccR-mediated regulation, which are induced by KDPG. RccR-mediated carbon metabolism pathways are colored in blue. *aceE*/*F*: pyruvate dehydrogenase subunits; *aceA*: isocitrate lyase; *glcB*: malate synthase G.

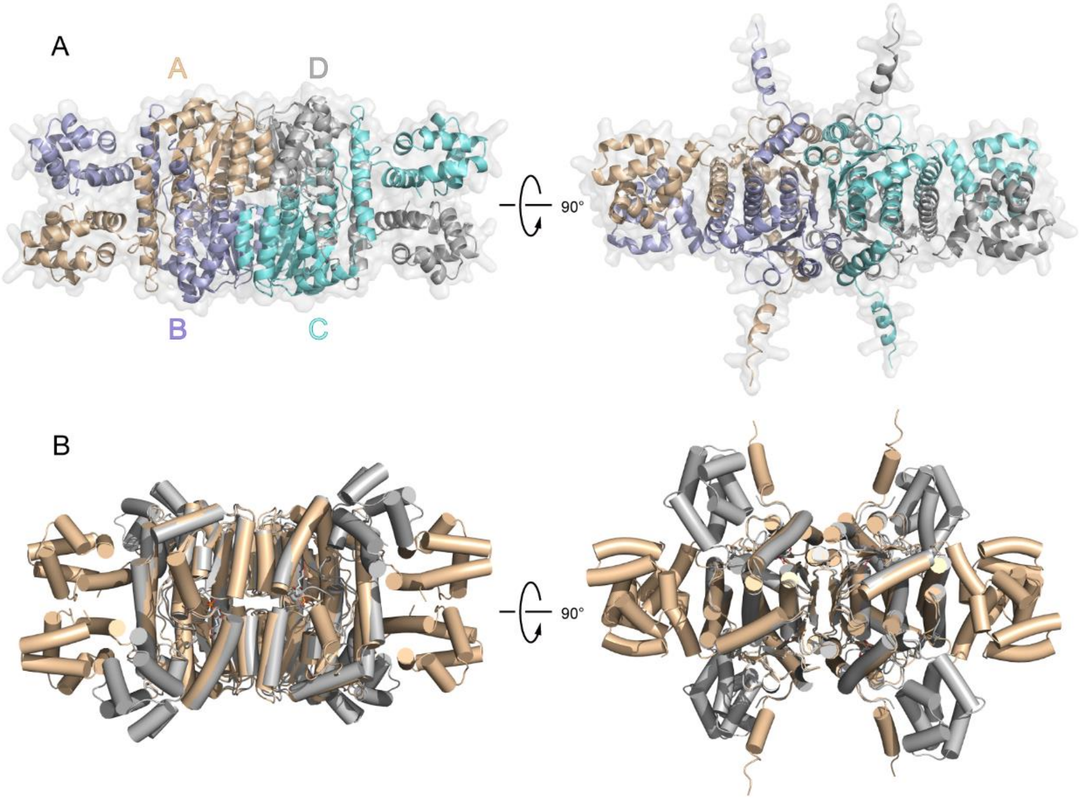

Figure S7. Structural characterizations of RccR.

A. The overall structure of apo-RccR predicted by ColabFold. The monomers (A-D) are marked in wheat, lightblue, aquamarine, and gray, respectively.

B. Structure overlay of apo-RccR and RccR/KDPG complex. The predicted apo-RccR is shown in wheat and the RccR/KDPG complex is shown in gray.

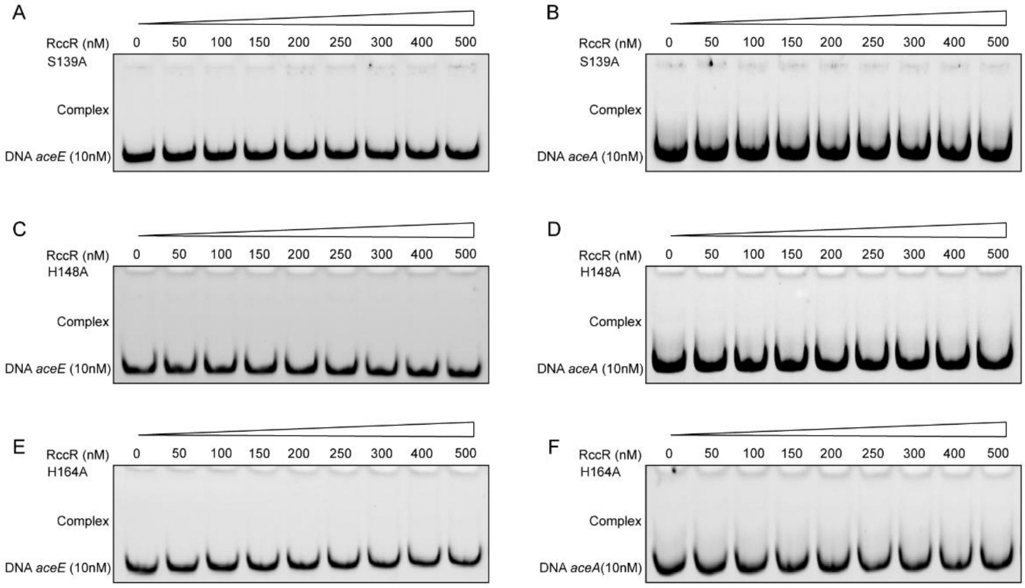

Figure S8. The EMSA analysis of the interaction between S139A, H148A, and H164A single mutant RccR proteins and the promoter DNA.

A and B. The EMSA of RccR-S139A protein and *aceE* (A) or *aceA* (B) promotor DNA.

C and D. The EMSA of RccR-H148A protein with *aceE* (C) or *aceA* (D) promotor DNA.

E and F. The EMSA of RccR-H164A protein with *aceE* (E) or *aceA* (F) promotor DNA.

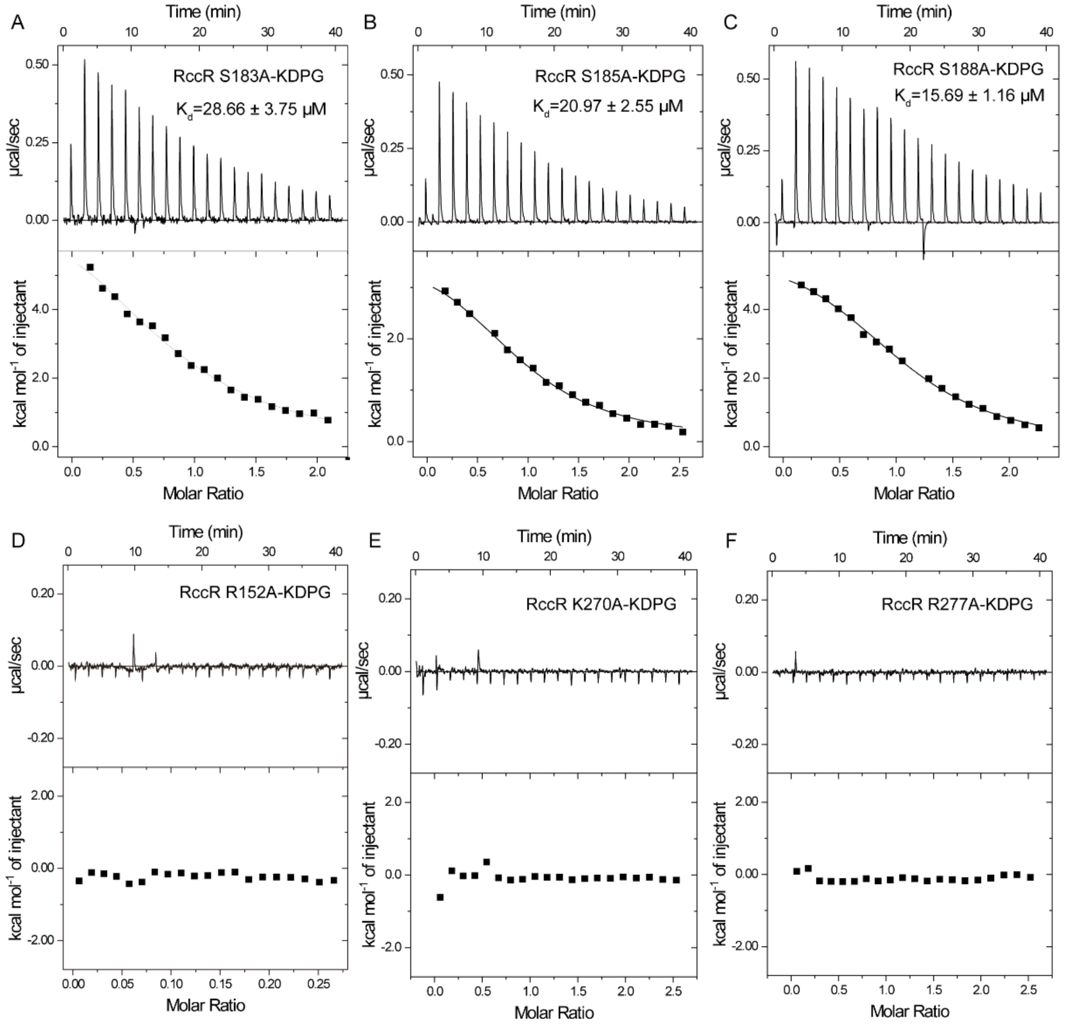

Figure S9. ITC assay of the binding affinities between KDPG and single mutant RccR proteins. K_d, the dissociation constant. The binding abilities of single mutant S183A (A), S185A (B), and S188A (C) proteins to KDPG decrease significantly, and single mutant R152A (D), K270A (E), and R277A (F) proteins showed no detectable binding affinities to KDPG.

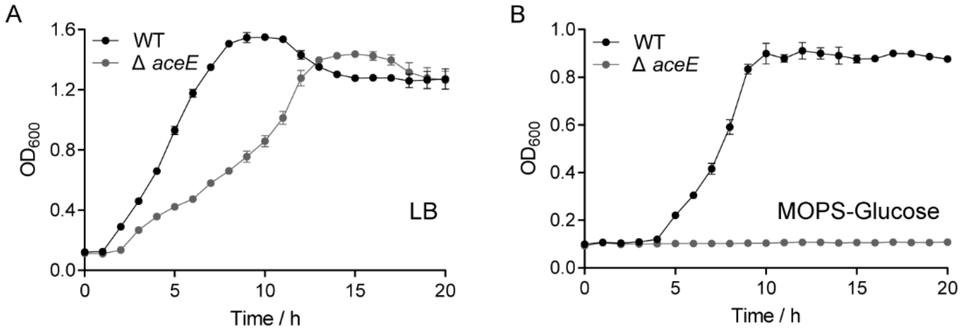

Figure S10. The growth curves of PAO1 WT and *aceE*-deletion strains in LB medium (A) and MOPS minimal medium supplemented with glucose (B). WT, wild-type.

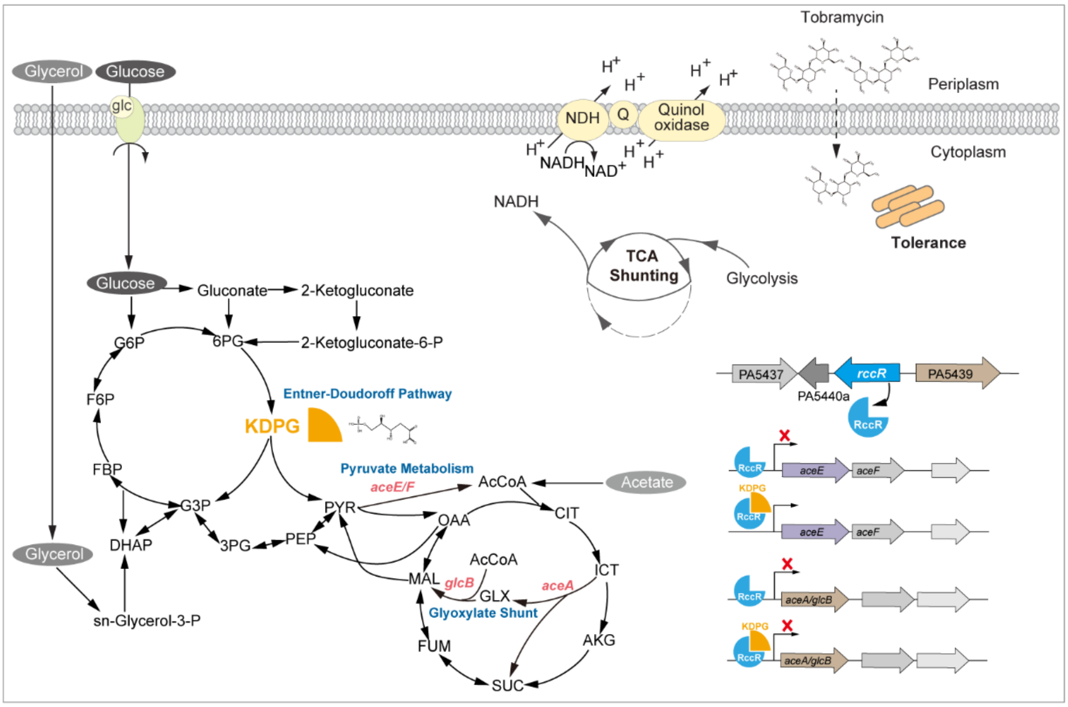

Figure S11. The schematic illustration of aminoglycoside antibiotic tolerance, the E-D pathway, pyruvate catabolism, the glyoxylate shunt, and RccR transcriptional regulation.

Table S1. Data collection, phasing, and refinement statistics of the structures of RccR/KDPG complex.

	RccR/KDPG
Wavelength (Å)	0.9792
Beamline[a]	BL18U1
Space group	I222
Cell dimensions	
a, b, c (Å)	78.138, 81.624, 137.888
α, β, γ (°)	90.00, 90.00, 90.00
Resolution (Å)	50-1.90
R_{merge} (%)	8.9 (65.2)[b]
I/σI	25.8 (2.4)
Completeness (%)	98 (93.1)
Redundancy	11.7 (8.7)
Refinement	
Resolution (Å)	26.01-1.90
Reflections (#)	26374
R_{work}/R_{free} (%)	20.79/24.26
Number of nonhydrogen Atoms (#)	
Protein	2124
Ligand	16
H_2O	161
Rmsds	
Bond lengths (Å)	0.015
Bond angle (°)	1.91
Average B factors ($Å^2$)	
Overall	33.10
Protein	32.80
Ligand	18.38

[a]Crystal data were collected at the BL18U1 beamlines of the National Facility for Protein Science Shanghai (NFPS) at Shanghai Synchrotron Radiation Facility.

[b]Statistics for the highest-resolution shell are shown in parentheses.

Table S2. Bacterial strains and plasmids used in this study.

Strains or plasmids	Description	Reference
Strains		
E.coli		
DH5α	F-φ80*lacZ* ΔM15 Δ(*lacZ*YA-argF) U169 *endA*1 *recA*1 *hsdR*17(rk-,mk+) *supE*44λ-thi-1 *gyrA*96 relA1 *phoA*	Lab stock
BL21 (DE3)	fhuA2 [lon] ompT gal (λ DE3) [dcm] ΔhsdS λ DE3 = λ sBamHIo ΔEcoRI-B int::(lacI::PlacUV5::T7 gene1) i21 Δnin5	Lab stock
P. aeruginosa		
PAO1 wild-type	Wild-type strain	Lab stock
PAO1_Δ*rccR*	PAO1 Δ*rccR*	This study
PAO1_Δ*rccR* + *rccR*	PAO1_Δ*rccR* mutation with *rccR* complementation	This study
PAO1_Δ*aceE*	PAO1 Δ*aceE*	This study
PAO1 + *aceA*	PAO1 overexpressing *aceA* gene	This study
PAO1 + *glcB*	PAO1 overexpressing *glcB* gene	This study
PAO1_Δ*rccR* + *rccR*-R152A	PAO1_Δ*rccR* mutation with *rccR*_R152A complementation	This study
PAO1_Δ*rccR* + *rccR*_S183A	PAO1_Δ*rccR* mutation with *rccR*_S183A complementation	This study
PAO1_Δ*rccR* + *rccR*_S185A	PAO1_Δ*rccR* mutation with *rccR*_S185A complementation	This study
PAO1_Δ*rccR* + *rccR*_S188A	PAO1_Δ*rccR* mutation with *rccR*_S188A complementation	This study
PAO1_Δ*rccR* + *rccR*_K270A	PAO1_Δ*rccR* mutation with *rccR*_K270A complementation	This study
PAO1_Δ*rccR* + *rccR*_R277A	PAO1_Δ*rccR* mutation with *rccR*_R277A complementation	This study
Plasmids		
pACRISPR-NN1-*rccR*	pACRISPR plasmid derivative, for genome editing in *P. aeruginosa*. Carbenicillin resistance.	This study
pACRISPR-NN2-*rccR*	pACRISPR plasmid derivative, for genome editing in *P. aeruginosa*. Carbenicillin resistance.	This study
pACRISPR-NN2mut-*rccR*	pACRISPR plasmid derivative, containing single mutation in repair template of *rccR*, for genome editing in *P. aeruginosa*. Carbenicillin resistance.	This study
pACRISPR-NN-*aceE*	pACRISPR plasmid derivative, for genome editing in *P. aeruginosa*. Carbenicillin resistance.	This study

pAK1900-*rpsl*-*aceA*	pAK1900 plasmid derivative, for overexpressing *aceA* in *P. aeruginosa*. Carbenicillin resistance.	This study
pAK1900-*rpsl*-*glcB*	pAK1900 plasmid derivative, for overexpressing *glcB* in *P. aeruginosa*. Carbenicillin resistance.	This study
pET28a-RccR-his	pET28a plasmid derivative, for expressing RccR wild-type protein. Kanamycin resistance.	This study
pET28a-RccR_S139A-his	pET28a plasmid derivative, for expressing RccR_S139A mutant protein. Kanamycin resistance.	This study
pET28a-RccR_H148A-his	pET28a plasmid derivative, for expressing RccR_H148A mutant protein. Kanamycin resistance.	This study
pET28a-RccR_R152A-his	pET28a plasmid derivative, for expressing RccR_R152A mutant protein. Kanamycin resistance.	This study
pET28a-RccR_H164A-his	pET28a plasmid derivative, for expressing RccR_H164A mutant protein. Kanamycin resistance.	This study
pET28a-RccR_S183A-his	pET28a plasmid derivative, for expressing RccR_S183A mutant protein. Kanamycin resistance.	This study
pET28a-RccR_S185A-his	pET28a plasmid derivative, for expressing RccR_S185A mutant protein. Kanamycin resistance.	This study
pET28a-RccR_S188A-his	pET28a plasmid derivative, for expressing RccR_S188A mutant protein. Kanamycin resistance.	This study
pET28a-RccR_K270A-his	pET28a plasmid derivative, for expressing RccR_K270A mutant protein. Kanamycin resistance.	This study
pET28a-RccR_R277A-his	pET28a plasmid derivative, for expressing RccR_R277A mutant protein. Kanamycin resistance.	This study

Table S3. Primers used in this study.

	Name	Sequence (5'-3')
rccR gene deletion and complementation	*rccR*-spacer-F	GTGGCTCGATCCATGAGAGCGACT
	rccR-spacer-R	AAACAGTCGCTCTCATGGATCGAG
	rccR-T1-F	CGAGCCCAGCCTGGCGCCGCCGGGAAGGCATAACG
	rccR-T1-R	CCCGGCGGCGCCAGGCTGGGCTCGATTCTCTGTAG
	rccR-T2-F	TTTTGAGATCTGTCCATACCCATGGTCTAGACGAGGCCGGCGATGGAGAGGT
	rccR-T2-R	TCTGAATGGCGGGAGTATGAAAAGTCTCGAGCTGGCCCTGCTGCCGCGCCAT
	rccR-T3-F	ATCAGGTTCTGCACGGTTTCC
	rccR-T3-R	TGAAGGACCTGGAGCCCTTCC
	com-*rccR*-spacer-F	GTGGACAGAGAATCGAGCCCAGCC
	com-*rccR*-spacer-R	AAACGGCTGGGCTCGATTCTCTGT
	com-*rccR*-T1-F	CCGAACTGGCGCATGGGGTCGGGGTCAGCGAGCCGACCATC
	com-*rccR*-T1-R	CCCCGACCCCATGCGCCAGTTCGGCCATGGAGCTG
	com-*rccR*-T2-F	TTTTGAGATCTGTCCATACCCATGGTCTAGACGAGGCCGGCGATGGAGAGGT
	com-*rccR*-T2-R	CTTCTGAATGGCGGGAGTATGAAAAGTCTCGAGCTGGCCCTGCTGCCGCGCCATGCG
aceE gene deletion	*aceE*-spacer-F	GTGGATGAAGTACCTGGAAAGCCG
	aceE-spacer-R	AAACCGGCTTTCCAGGTACTTCAT
	aceE-T1-F	TTTCCGCCCTGGAGCAAGCCTTCGCTCCATACTCCCCTCGGCA
	aceE-T1-R	TGGAGCGAAGGCTTGCTCCAGGGCGGAAAG
	aceE-T2-F	TTTTGAGATCTGTCCATACCCATGGTCTAGATTCCAGCAGCATCGCCGAGTCG
	aceE-T2-R	TCTGAATGGCGGGAGTATGAAAAGTCTCGAGCGATGTCCGGGACCTTGATGTCCTG
	aceE-T3-F	TTGCTGGCGTGTGCGGAAAC
	aceE-T3-R	GACCTTGATCGAGACGCTTT
rccR gene mutation	S139A-F	TTCGGCGCTGCCGGCGCGGTGGCCTCGGATGCCCA

	S139A-R	CCACCGCGCCGGCAGCGCCGAAGCCGTAG AACTCCA
	S183A-F	ATCTGCATTGCCCAGTCGGGTCGCTCGAAG
	S183A-R	ACCCGACTGGGCAATGCAGATGGCCACGTC GC
	S185A-F	CATTTCCCAGGCGGGTCGCTCGAAGGACCT G
	S185A-R	CGAGCGACCCGCCTGGGAAATGCAGATGG CC
	S188A-F	CGGGTCGCGCGAAGGACCTGCTGATCACC G
	S188A-R	GGTCCTTCGCGCGACCCGACTGGGAAATGC
	H148A-F	ATGCCCAGGCCAAGTTCTTCCGCCTGCTGC TGTCGGCG
	H148A-R	GAAGAACTTGGCCTGGGCATCCGAGGCCAC CGCG
	H164A-F	CTCCGACCCGGCCATGCAGGCGATGTCGG CGGTGACC
	H164A-R	CGCCTGCATGGCCGGGTCGGAGTAGGCCG CCGCC
	R152A-F	GTTCTTCGCCCTGCTGCTGTCGGCGGCGG CCTA
	R152A-R	CGACAGCAGCAGGGCGAAGAACTTGTGCT GGGCATCCGAGGCCAC
	K270A-F	AGAGCGTCGCGCGCAGCCTGCGCAGCCTG CGCCTG
	K270A-R	GCGCAGGCTGCGCGCGACGCTCTTGAGGT GGTTGACCAGGTCCGG
	R277A-F	GCAGCCTGGCCCTGTCGCCGAAGGTGATG AAGAACCAGGAAG
	R277A-R	CTTCGGCGACAGGGCCAGGCTGCGCAGGC TGCGCTTGAC
EMSA	*aceE*-EMSA-200F	ACATCGGGCCTTATAGCCTG
	aceE-EMSA-200R	GGCTTGCTCCAGGGCGGAAA
	aceA-EMSA-500F	AAACATCTCGACCAGACAAGAC
	aceA-EMSA-500R	GGTCAATCCTTCAAGAAATGT
	glcB-EMSA-200F	TGGGCTCGTGATAAATCGTG
	glcB-EMSA-200R	TGTTTGCCTCACTCTGCTCT
qRT-PCR	*gyrB*-RT-F1	GCCGGTTACTGCAGCGAAATC

gyrB-RT-R1	ATGATCACTTCCGCCGCAGAA
aceE-RT-F1	GAACAAGCACGACCCGGACC
aceE-RT-R1	CCCTGGAAGAACACCAGGTCG
aceF-RT-F1	CCGACAAGGCCAGCATGGAAA
aceF-RT-R1	GCCTTCCACTTCCAGCTCGA
aceA-RT-F1	GCGAGCTGGACCTGCTGTT
aceA-RT-R1	GATGATCGGCACCACGTGGG
glcB-RT-F1	TCTGCCGACACGTTCTGGACC
glcB-RT-R1	TGGTGCCAGCCGTCGATCTT

Table S4. Transcriptome sequencing results in this study.

Table S4.1 Transcriptome sequencing results of stationary phase strains.

Gene ID	Gene name	Gene description	Log_2FC	*P*-value
Transporters				
PA0184	PA0184	ABC transporter ATP-binding protein	-8.410	4.23E-08
PA0185	PA0185	ABC transporter permease	-8.838	4.92E-09
PA0186	PA0186	ABC transporter	-7.845	5.38E-07
PA3445	PA3445	hypothetical protein	-1.908	1.21E-01
PA3449	PA3449	hypothetical protein	-1.166	3.58E-01
PA3442	PA3442	ABC transporter ATP-binding subunit	-2.363	8.65E-05
PA3443	PA3443	ABC transporter permease	-2.478	5.27E-05
PA3935	*tauD*	taurine dioxygenase	-1.058	1.14E-02
PA3936	PA3936	taurine ABC transporter permease	-1.082	4.59E-02
PA3937	PA3937	taurine ABC transporter ATP-binding protein	-1.846	2.55E-03
PA3938	PA3938	taurine-binding protein	-1.365	3.13E-03
PA0203	PA0203	ABC transporter	-7.349	4.91E-06
PA0204	PA0204	ABC transporter permease	-7.039	1.82E-05
PA0205	PA0205	ABC transporter permease	-7.100	1.41E-05
PA0206	PA0206	spermidine/putrescine ATP-binding protein	-7.064	1.65E-05
PA2407	PA2407	adhesion protein	-1.304	5.62E-06
PA2408	PA2408	ABC transporter ATP-binding protein	-1.445	5.12E-04
PA2409	PA2409	ABC transporter permease	-1.155	1.01E-02
PA1633	*kdpA*	potassium-transporting ATPase subunit A	-1.200	9.35E-05
PA1634	*kdpB*	potassium-transporting ATPase subunit B	-1.582	3.65E-05
PA1635	*kdpC*	potassium-transporting ATPase subunit C	-1.608	2.81E-03
PA3187	PA3187	ABC transporter ATP-binding protein	1.032	1.59E-03
PA4222	PA4222	ABC transporter ATP-binding protein	-2.698	1.25E-14
PA4223	PA4223	ABC transporter ATP-binding protein	-2.590	3.74E-17
PA3188	PA3188	sugar ABC transporter permease	1.127	1.81E-02
PA3190	PA3190	sugar ABC transporter substrate-binding protein	1.250	4.82E-02

PA0166	PA0166	transporter	-7.501	2.56E-06
PA0188	PA0188	hypothetical protein	-7.448	3.13E-06
PA0189	PA0189	porin	-7.624	1.49E-06
PA0192	PA0192	TonB-dependent receptor	-7.058	1.74E-05
PA0197	*tonB2*	transporter TonB	-5.713	2.95E-03
PA0198	*exbB1*	transporter ExbB	-8.895	4.73E-09
PA0199	*exbD1*	biopolymer transport protein ExbD	-7.827	5.86E-07
PA1286	PA1286	major facilitator superfamily transporter	-1.559	1.35E-02
PA2398	*fpvA*	ferripyoverdine receptor	-2.366	3.05E-24
PA4218	PA4218	transporter	-3.224	1.99E-04
PA4221	*fptA*	Fe(III)-pyochelin outer membrane receptor	-3.358	1.34E-29
PA4710	*phuR*	heme/hemoglobin uptake outer membrane receptor PhuR	-1.513	1.34E-07
Metabolic				
PA0195	*pntAA*	NAD(P) transhydrogenase subunit alpha	-14.560	1.54E-23
PA0195.1	*pntAB*	NAD(P) transhydrogenase subunit alpha	-10.055	1.14E-11
PA0196	*pntB*	pyridine nucleotide transhydrogenase subunit beta	-10.653	1.43E-13
PA2634	*aceA*	isocitrate lyase	2.403	2.04E-27
PA4640	*mqoB*	malate:quinone oxidoreductase	-1.631	1.81E-19
PA5015	*aceE*	pyruvate dehydrogenase subunit E1	4.172	1.19E-109
PA5016	*aceF*	dihydrolipoamide acetyltransferase	3.872	1.18E-103
PA5192	*pckA*	phosphoenolpyruvate carboxykinase	1.089	1.97E-06
PA0182	PA0182	3-ketoacyl-ACP reductase	-8.806	5.72E-09
PA4888	*desB*	acyl-CoA desaturase	1.507	1.31E-08
PA0190	PA0190	acid phosphatase	-9.894	2.49E-11
PA0168	PA0168	hypothetical protein	-8.883	3.93E-09
PA0183	*atsA*	arylsulfatase	-9.685	7.17E-11
PA0193	PA0193	hypothetical protein	-8.363	4.84E-08
PA0201	PA0201	hypothetical protein	-12.120	1.36E-11
PA0202	PA0202	amidase	-7.427	3.93E-06
PA1001	*phnA*	anthranilate synthase component I	-1.845	1.20E-15
PA1002	*phnB*	anthranilate synthase component II	-1.075	3.88E-05

PA2413	*pvdH*	diaminobutyrate--2-oxoglutarate aminotransferase	-2.087	1.86E-13
PA2385	*pvdQ*	acyl-homoserine lactone acylase PvdQ	-3.300	3.98E-17
PA2386	*pvdA*	L-ornithine N5-oxygenase	-3.482	9.10E-36
PA2393	PA2393	dipeptidase	-2.840	1.31E-09
PA2424	*pvdL*	peptide synthase	-2.498	7.82E-27
PA4212	*phzC1*	phenazine biosynthesis protein PhzC	-1.352	7.05E-06
PA4224	*pchG*	pyochelin biosynthetic protein PchG	-3.506	9.33E-26
PA4225	*pchF*	pyochelin synthetase	-3.626	1.18E-05
PA4226	*pchE*	dihydroaeruginoic acid synthetase	-3.563	4.55E-08
PA4228	*pchD*	2%2C3-dihydroxybenzoate-AMP ligase	-4.790	1.41E-65
PA4229	*pchC*	pyochelin biosynthetic protein PchC	-4.588	2.19E-33
PA4230	*pchB*	isochorismate-pyruvate lyase	-3.911	4.58E-20
PA4231	*pchA*	salicylate biosynthesis isochorismate synthase	-4.055	1.62E-38
PA2394	*pvdN*	pyoverdine biosynthesis protein PvdN	-2.610	2.71E-10
PA2395	*pvdO*	pyoverdine biosynthesis protein PvdO	-2.696	2.96E-09
PA2397	*pvdE*	pyoverdine biosynthesis protein PvdE	-2.210	1.74E-08
PA2425	*pvdG*	pyoverdine biosynthesis protein PvdG	-3.111	3.41E-06
PA0187	PA0187	hypothetical protein	-7.539	2.12E-06
PA0194	PA0194	hypothetical protein	-8.052	2.05E-07
PA1899	*phzA2*	phenazine biosynthesis protein PhzA	-1.667	1.38E-01
PA4211	*phzB1*	phenazine biosynthesis protein	-1.182	6.78E-04
PA2512	*antA*	anthranilate dioxygenase large subunit	-1.485	5.82E-07
PA2513	*antB*	anthranilate dioxygenase small subunit	-1.997	1.52E-04
PA2514	*antC*	anthranilate dioxygenase reductase	-1.095	1.70E-02
Bacterial chemotaxis				
PA0173	PA0173	chemotaxis response regulator protein	-10.453	1.59E-12

PA0174	PA0174	hypothetical protein	-9.131	1.16E-09
PA0175	PA0175	chemotaxis protein methyltransferase	-12.862	1.59E-18
PA0176	*aer2*	aerotaxis transducer Aer2	-14.287	2.76E-23
PA0177	PA0177	purine-binding chemotaxis protein	-13.032	3.92E-19
PA0178	PA0178	two-component sensor	-13.247	2.85E-37
PA0179	PA0179	two-component response regulator	-14.862	1.85E-24
PA0180	*cttP*	trichloroethylene chemotactic transducer CttP	-14.590	1.22E-23
Quorum sensing				
PA0996	*pqsA*	anthranilate--CoA ligase	-3.583	6.27E-44
PA0997	*pqsB*	hypothetical protein	-4.058	6.48E-56
PA0998	*pqsC*	hypothetical protein	-3.219	1.77E-34
PA0999	*pqsD*	3-oxoacyl-ACP synthase	-2.317	2.84E-25
PA1000	*pqsE*	thioesterase PqsE	-2.016	1.51E-15
Regulator protein				
PA0167	PA0167	transcriptional regulator	-10.651	4.43E-13
PA0181	PA0181	transcriptional regulator	-10.720	3.07E-13
PA0191	PA0191	transcriptional regulator	-8.303	7.22E-08
PA0207	PA0207	transcriptional regulator	-1.880	2.14E-10
PA2785	PA2785	hypothetical protein	-3.466	2.57E-01
Biofilm formation				
PA0169	PA0169	hypothetical protein	-10.481	1.34E-12
PA0170	PA0170	hypothetical protein	-8.614	2.21E-08
PA0171	PA0171	hypothetical protein	-7.804	1.31E-07
PA0172	PA0172	hypothetical protein	-10.214	4.65E-12
Other genes				
PA0165	PA0165	hypothetical protein	-8.991	2.50E-09
PA0200	PA0200	hypothetical protein	-12.397	2.44E-17
PA0691	PA0691	hypothetical protein	1.470	3.28E-01
PA1168	PA1168	hypothetical protein	-1.697	6.09E-04
PA1300	PA1300	ECF subfamily sigma-70 factor	-1.603	5.68E-03
PA1301	PA1301	transmembrane sensor	-1.462	7.73E-03
PA1956	PA1956	hypothetical protein	-1.997	3.25E-02
PA2036	PA2036	hypothetical protein	1.821	7.39E-02
PA2311	PA2311	hypothetical protein	-1.251	2.45E-01
PA2384	PA2384	hypothetical protein	-1.474	3.86E-02
PA2406	PA2406	hypothetical protein	-1.470	2.27E-04
PA2411	PA2411	thioesterase	-2.607	1.93E-16

PA2412	PA2412	hypothetical protein	-1.722	7.12E-03
PA2426	*pvdS*	extracytoplasmic-function sigma-70 factor	-3.258	8.78E-10
PA2427	PA2427	hypothetical protein	-1.627	1.51E-02
PA3444	PA3444	alkanesulfonate monooxygenase	-2.337	5.72E-02
PA3530	PA3530	hypothetical protein	-1.546	2.38E-02
PA4060	PA4060	hypothetical protein	5.681	3.11E-03
PA4219	PA4219	hypothetical protein	-2.680	8.15E-13
PA4469	PA4469	hypothetical protein	-1.628	1.77E-04
PA4635	PA4635	hypothetical protein	-1.838	3.13E-05
PA4883	PA4883	hypothetical protein	-2.094	6.78E-02
PA5083	PA5083	hypothetical protein	1.496	3.27E-01
PA5404	PA5404	hypothetical protein	-2.752	1.03E-01
PA5440a	PA5440a	hypothetical protein	2.837	3.32E-36

Table S4.2 Transcriptome sequencing results of logarithmic phase strains.

Gene ID	Gene name	Gene description	Log_2FC	*P*-value
Transporters				
PA0184	PA0184	ABC transporter ATP-binding protein	-9.020	1.84E-09
PA0185	PA0185	ABC transporter permease	-7.440	6.76E-07
PA0186	PA0186	ABC transporter	-7.548	1.78E-06
PA3445	PA3445	hypothetical protein	-1.511	1.16E-03
PA3449	PA3449	hypothetical protein	-1.709	2.22E-01
PA0203	PA0203	ABC transporter	-7.640	1.25E-06
PA0204	PA0204	ABC transporter permease	-7.276	5.83E-06
PA0205	PA0205	ABC transporter permease	-6.790	4.67E-05
PA0206	PA0206	spermidine/putrescine ATP-binding protein	-7.356	4.88E-06
PA1873	PA1873	hypothetical protein	1.267	1.22E-01
PA2407	PA2407	adhesion protein	-2.127	7.78E-07
PA2408	PA2408	ABC transporter ATP-binding protein	-1.719	1.31E-02
PA2409	PA2409	ABC transporter permease	-1.695	7.42E-03
PA0166	PA0166	transporter	-7.329	4.61E-06
PA0188	PA0188	hypothetical protein	-6.466	1.50E-04
PA0189	PA0189	porin	-7.092	1.21E-05
PA0192	PA0192	TonB-dependent receptor	-7.346	4.10E-06
PA0197	*tonB2*	transporter TonB	-5.839	1.45E-03
PA0198	*exbB1*	transporter ExbB	-9.185	8.32E-10
PA0199	*exbD1*	biopolymer transport protein ExbD	-6.787	4.42E-05
PA5167	PA5167	C4-dicarboxylate-binding protein	-1.517	8.10E-08
PA5168	PA5168	dicarboxylate transporter	-1.693	3.29E-06
PA5169	PA5169	C4-dicarboxylate transporter	-2.016	2.66E-10
PA0166	PA0166	transporter	-7.329	4.61E-06
PA0188	PA0188	hypothetical protein	-6.466	1.50E-04
PA0189	PA0189	porin	-7.092	1.21E-05
PA0192	PA0192	TonB-dependent receptor	-7.346	4.10E-06
PA0197	*tonB2*	transporter TonB	-5.839	1.45E-03
PA0198	*exbB1*	transporter ExbB	-9.185	8.32E-10
PA0199	*exbD1*	biopolymer transport protein ExbD	-6.787	4.42E-05
PA5167	PA5167	C4-dicarboxylate-binding protein	-1.517	8.10E-08
PA5168	PA5168	dicarboxylate transporter	-1.693	3.29E-06
PA5169	PA5169	C4-dicarboxylate transporter	-2.016	2.66E-10
Metabolic				
PA0195	*pntAA*	NAD(P) transhydrogenase subunit alpha	-12.179	7.80E-17

PA0195.1	*pntAB*	NAD(P) transhydrogenase subunit alpha	-8.551	1.79E-08
PA0196	*pntB*	pyridine nucleotide transhydrogenase subunit beta	-10.734	2.89E-13
PA0482	*glcB*	malate synthase G	1.937	7.36E-21
PA2261	PA2261	2-ketogluconate kinase	1.202	1.21E-04
PA2634	*aceA*	isocitrate lyase	4.741	1.02E-134
PA4640	*mqoB*	malate:quinone oxidoreductase	-1.181	1.53E-08
PA5015	*aceE*	pyruvate dehydrogenase subunit E1	1.948	1.88E-21
PA5016	*aceF*	dihydrolipoamide acetyltransferase	2.553	5.69E-42
PA5192	*pckA*	phosphoenolpyruvate carboxykinase	1.092	1.78E-07
PA0520	*nirQ*	denitrification regulatory protein NirQ	1.364	1.36E-01
PA0521	PA0521	cytochrome C oxidase subunit	1.210	2.92E-01
PA2664	*fhp*	flavohemoprotein	1.849	2.91E-01
PA3872	*narI*	respiratory nitrate reductase subunit gamma	4.108	7.81E-03
PA3873	*narJ*	respiratory nitrate reductase subunit delta	4.089	7.80E-03
PA3874	*narH*	respiratory nitrate reductase subunit beta	3.931	1.10E-02
PA3875	*narG*	respiratory nitrate reductase subunit alpha	3.667	1.68E-02
PA3876	*narK2*	nitrite extrusion protein 2	2.312	1.01E-01
PA0182	PA0182	3-ketoacyl-ACP reductase	-9.861	2.82E-11
PA0190	PA0190	acid phosphatase	-9.601	1.08E-10
PA0164	PA0164	gamma-glutamyltranspeptidase	-1.276	1.14E-07
PA0168	PA0168	hypothetical protein	-10.310	2.72E-12
PA0183	*atsA*	arylsulfatase	-10.864	1.66E-13
PA0193	PA0193	hypothetical protein	-8.989	2.94E-09
PA0201	PA0201	hypothetical protein	-12.780	2.26E-18
PA0202	PA0202	amidase	-7.836	5.57E-07
PA2193	*hcnA*	hydrogen cyanide synthase subunit HcnA	-1.520	3.02E-07
PA2194	*hcnB*	hydrogen cyanide synthase subunit HcnB	-1.276	9.86E-07
PA4212	*phzC1*	phenazine biosynthesis protein PhzC	-5.371	3.15E-10
PA5023	PA5023	hypothetical protein	1.883	1.66E-01
PA0187	PA0187	hypothetical protein	-6.875	2.97E-05
PA2664	*fhp*	flavohemoprotein	1.849	2.91E-01

PA3872	*narI*	respiratory nitrate reductase subunit gamma	4.108	7.81E-03
PA3873	*narJ*	respiratory nitrate reductase subunit delta	4.089	7.80E-03
PA3874	*narH*	respiratory nitrate reductase subunit beta	3.931	1.10E-02
PA3875	*narG*	respiratory nitrate reductase subunit alpha	3.667	1.68E-02
PA3876	*narK2*	nitrite extrusion protein 2	2.312	1.01E-01
PA0182	PA0182	3-ketoacyl-ACP reductase	-9.861	2.82E-11
PA0190	PA0190	acid phosphatase	-9.601	1.08E-10
PA0164	PA0164	gamma-glutamyltranspeptidase	-1.276	1.14E-07
PA0168	PA0168	hypothetical protein	-10.310	2.72E-12
PA0183	*atsA*	arylsulfatase	-10.864	1.66E-13
PA0193	PA0193	hypothetical protein	-8.989	2.94E-09
PA0201	PA0201	hypothetical protein	-12.780	2.26E-18
PA0202	PA0202	amidase	-7.836	5.57E-07
PA2193	*hcnA*	hydrogen cyanide synthase subunit HcnA	-1.520	3.02E-07
PA2194	*hcnB*	hydrogen cyanide synthase subunit HcnB	-1.276	9.86E-07
PA4212	*phzC1*	phenazine biosynthesis protein PhzC	-5.371	3.15E-10
PA5023	PA5023	hypothetical protein	1.883	1.66E-01
PA0187	PA0187	hypothetical protein	-6.875	2.97E-05
PA0190	PA0190	acid phosphatase	-9.601	1.08E-10
PA0164	PA0164	gamma-glutamyltranspeptidase	-1.276	1.14E-07
PA0168	PA0168	hypothetical protein	-10.310	2.72E-12
PA0183	*atsA*	arylsulfatase	-10.864	1.66E-13
PA0193	PA0193	hypothetical protein	-8.989	2.94E-09
PA0201	PA0201	hypothetical protein	-12.780	2.26E-18
PA0202	PA0202	amidase	-7.836	5.57E-07
PA2193	*hcnA*	hydrogen cyanide synthase subunit HcnA	-1.520	3.02E-07
PA2194	*hcnB*	hydrogen cyanide synthase subunit HcnB	-1.276	9.86E-07
PA4212	*phzC1*	phenazine biosynthesis protein PhzC	-5.371	3.15E-10
PA5023	PA5023	hypothetical protein	1.883	1.66E-01
PA0187	PA0187	hypothetical protein	-6.875	2.97E-05
PA0194	PA0194	hypothetical protein	-8.141	1.78E-07
PA3870	*moaA1*	molybdenum cofactor biosynthesis protein A	4.049	7.68E-03

PA3914	*moeA1*	molybdenum cofactor biosynthesis protein A	1.883	1.75E-01
Bacterial chemotaxis				
PA0173	*cheB*	chemotaxis response regulator protein	-7.161	9.48E-06
PA0174	*cheD*	hypothetical protein	-6.003	9.09E-04
PA0175	*cheR*	chemotaxis protein methyltransferase	-9.404	2.81E-10
PA0176	*aer2*	aerotaxis transducer Aer2	-10.204	2.47E-12
PA0177	*cheW*	purine-binding chemotaxis protein	-9.089	1.41E-09
PA0178	*cheA*	two-component sensor	-11.678	1.50E-15
PA0179	PA0179	two-component response regulator	-10.468	1.22E-12
PA0180	*cttP*	trichloroethylene chemotactic transducer CttP	-12.770	2.33E-18
Quorum sensing				
PA0996	*pqsA*	probable coenzyme A ligase	-1.564	8.96E-03
PA0997	*pqsB*	Homologous to beta-keto-acyl-acyl-carrier protein synthase	-1.386	4.48E-03
PA0998	*pqsC*	Homologous to beta-keto-acyl-acyl-carrier protein synthase	-1.386	2.69E-03
Regulator protein				
PA0194	PA0194	hypothetical protein	-8.141	1.78E-07
PA3870	*moaA1*	molybdenum cofactor biosynthesis protein A	4.049	7.68E-03
PA3914	*moeA1*	molybdenum cofactor biosynthesis protein A	1.883	1.75E-01
Bacterial chemotaxis				
PA0173	*cheB*	chemotaxis response regulator protein	-7.161	9.48E-06
PA0174	*cheD*	hypothetical protein	-6.003	9.09E-04
PA0175	*cheR*	chemotaxis protein methyltransferase	-9.404	2.81E-10
PA0176	*aer2*	aerotaxis transducer Aer2	-10.204	2.47E-12
PA0177	*cheW*	purine-binding chemotaxis protein	-9.089	1.41E-09
PA0178	*cheA*	two-component sensor	-11.678	1.50E-15
PA0179	PA0179	two-component response regulator	-10.468	1.22E-12

PA0180	*cttP*	trichloroethylene chemotactic transducer CttP	-12.770	2.33E-18
Quorum sensing				
PA0996	*pqsA*	probable coenzyme A ligase	-1.564	8.96E-03
PA0997	*pqsB*	Homologous to beta-keto-acyl-acyl-carrier protein synthase	-1.386	4.48E-03
PA0998	*pqsC*	Homologous to beta-keto-acyl-acyl-carrier protein synthase	-1.386	2.69E-03
Regulator protein				
PA0167	PA0167	transcriptional regulator	-12.687	3.79E-18
PA0181	PA0181	transcriptional regulator	-12.012	2.12E-16
PA0191	PA0191	transcriptional regulator	-8.498	2.25E-08
PA2663	*ppyR*	psl and pyoverdine operon regulator PpyR	1.404	2.65E-01
Biofilm formation				
PA0169	PA0169	hypothetical protein	-12.158	1.32E-16
PA0170	PA0170	hypothetical protein	-9.895	2.48E-11
PA0171	PA0171	hypothetical protein	-9.919	1.02E-11
PA0172	PA0172	hypothetical protein	-11.537	3.72E-15
Bacterial secretion system				
PA2672	PA2672	type II secretion system protein	1.548	1.75E-01
PA2674	PA2674	type II secretion system protein	1.772	1.71E-01
Chaperonin				
PA3871	PA3871	PpiC-type peptidyl-prolyl cis-trans isomerase	4.182	6.56E-03
PA4385	*groEL*	molecular chaperone GroEL	1.319	3.80E-08
PA4386	*groES*	co-chaperonin GroES	1.688	7.26E-11
Other genes				
PA0165	PA0165	hypothetical protein	-11.168	1.84E-14
PA0200	PA0200	hypothetical protein	-12.445	1.35E-17
PA0277	PA0277	hypothetical protein	-1.592	2.70E-09
PA0665	PA0665	iron-sulfur cluster insertion protein ErpA	1.649	6.68E-02
PA0675	*vreI*	ECF sigma factor VreI	-1.696	7.21E-02
PA0819	PA0819	hypothetical protein	-1.520	2.25E-02
PA1168	PA1168	hypothetical protein	3.343	2.90E-02
PA1953	PA1953	hypothetical protein	-2.148	4.02E-01
PA2140	PA2140	metallothionein	-2.259	5.79E-01
PA2311	PA2311	hypothetical protein	1.463	2.05E-01

PA2405	PA2405	hypothetical protein	-1.587	1.98E-02
PA2406	PA2406	hypothetical protein	-1.914	9.19E-03
PA2635	PA2635	hypothetical protein	1.816	3.48E-11
PA2791	PA2791	hypothetical protein	-1.689	2.84E-01
PA2845	PA2845	hypothetical protein	1.313	1.86E-02
PA2916	PA2916	hypothetical protein	-1.836	9.53E-02
PA3428	PA3428	hypothetical protein	1.674	6.06E-02
PA3519	PA3519	hypothetical protein	1.419	9.36E-02
PA3530	PA3530	hypothetical protein	1.397	3.08E-01
PA3774	PA3774	acetylpolyamine aminohydrolase	1.845	5.17E-02
PA4087	PA4087	hypothetical protein	1.556	6.45E-02
PA4099	PA4099	hypothetical protein	1.210	2.47E-01
PA5275	PA5275	frataxin-like protein	1.602	8.53E-02
PA5440a	PA5440a	hypothetical protein	1.683	1.59E-11
